# Pain Assessment in Oral Medicine through Its Different Dimensions: A Comprehensive Review

**DOI:** 10.3390/dj11110246

**Published:** 2023-10-24

**Authors:** Andrea Scribante, Matteo Pellegrini, Federica Pulicari, Martina Ghizzoni, Francesco Paolo Modugno, Francesco Spadari

**Affiliations:** 1 Section of Dentistry, Department of Clinical, Surgical, Diagnostic and Pediatric Sciences, University of Pavia, 27100 Pavia, Italy; martina.ghizzoni01@universitadipavia.it (M.G.); francescopaolo.modugno02@universitadipavia.it (F.P.M.); 2 Maxillo-Facial Surgery and Dental Unit, Fondazione IRCCS Cà Granda Ospedale Maggiore Policlinico, 20122 Milan, Italy; federica.pulicari@studenti.unimi.it (F.P.); francesco.spadari@unimi.it (F.S.); 3 Department of Biomedical, Surgical and Dental Sciences, University of Milan, Via della Commenda 10, 20122 Milan, Italy

**Keywords:** anxiety, dentistry, depression, oral medicine, orofacial pain, dental pain, oral pathology, pain assessment, quality of life, sleep disorders

## Abstract

Orofacial pain is a complex experience made up of different features and involving various aspects of life. It has demonstrated a connection, especially when chronic, with conditions such as anxiety, depression, and sleep disorders, through paths that still have not been completely clarified. A deep understanding of orofacial pain and its impact on an individual’s life is critical for planning accurate diagnostic and therapeutic approaches. This review seeks to provide a comprehensive overview of the components constituting the pain experience, its implications in an individual’s life, the different tools for multidimensional pain assessment, and the specific applications for each tool. A comprehensive review was performed using the PubMed, Scopus, and Web of Science electronic databases. Ninety-five studies, including observational studies, clinical trials, case–control studies, and case reports, were included and analyzed in this review. Orofacial pain assessment exploits several methods, ranging from clinical evaluation to rating scales, questionnaires, and daily diaries. The choice of the correct instrument requires an evaluation of the type of pain experienced, of the patient’s characteristics and abilities to complete particular tasks, and finally, of the assessment tool features.

## 1. Introduction

Pain assessment is a clinical procedure with two primary functions: discriminative, to evaluate a precise stage in a patient or a single patient in a group (for example, to make a diagnosis), or evaluative, in order to assess the evolution of a patient’s medical or dental condition. Additionally, this kind of evaluation allows for a better understanding of the patient’s symptomatology, a prerequisite for gaining their full confidence [1]. Lastly, pain assessment can hold predictive value concerning the course of the disease, the degree of disability, the usage of therapeutic aids, and the social, economic, and work-related consequences. Acquiring this information is fundamental to establishing a series of non-pharmacological therapeutic regimens (such as relaxation techniques and cognitive strategies) [1,2]. Assessment tools can be distinguished into two groups: the first includes generic instruments, which are applicable to a wide variety of populations since they cover the complete spectrum of function, disability, and distress relevant to quality of life; the second includes specific instruments, which are built to focus on specific aspects of health status related to a particular disease (e.g., chronic lung disease, rheumatoid arthritis, or burning mouth syndrome), a population of patients (e.g., children, the elderly, or cognitively impaired subjects), a specific function (e.g., emotions, the ability to feed, speech, or sexual function), or a specific condition (e.g., pain) [3].

Any assessment tool must possess specific features that enable their application, the main ones being validity, reliability, and sensitivity. Validity indicates the capacity to measure the chosen quantity; valid rating scales aid in collecting higher-quality data with greater comparability, reducing effort and increasing the credibility of the data [4]. Validity can be categorized into content validity (the adequacy of the instrument to measure the chosen quantity, considering all the parameters that are important both for the patient and for the clinician), criterion-related validity (the ability to reflect the real clinical condition of the patients in the results), construct validity (the capacity to include all the variables important for the study of the chosen quantity), discriminatory validity (the ability to find clinically relevant differences among patients), and, lastly, convergent validity (the degree of correlation between the tool’s measures and other clinical measures) [4]. There are several ways to assess the validity of a questionnaire, such as comparing the results obtained with biological monitoring, personal exposure monitoring, or historical records [3].

Reliability indicates a low sensitivity to random error; when an instrument is reliable, it means that the results will be repeatable (always concerning the passing of time, changing symptomatology, and altering environment). Reliability is usually distinguished by internal consistency (the degree of inter-correlation among the items belonging to the same subscales and the items not belonging to the same subscales) and reproducibility (the stability of the tool’s results when the tests are repeated) [4].

The last important feature of a rating scale, particularly when used for evaluative purposes, is sensitivity, namely, the probability of encountering a clinically relevant change over time [3].

Pain assessment tools can be distinguished as subjective or objective tools: the first are usually in the form of rating scales, questionnaires, or pain diaries; the latter exploit technologies such as magnetic resonance and electroencephalography. The subjective methods have been considered the “gold standard” for pain assessment for a long time, showing limited accuracy and utility under certain circumstances: psychological (such as pain catastrophizing) or environmental factors may cause underestimation of the pain phenomenon; on the contrary, personality traits like feeling shame in showing vulnerability may cause underestimation. Lastly, the way the medical professional asks about the pain may cause bias [1,5].

The most common objective methods are neuroimaging techniques, such as functional magnetic resonance imaging (fMRI), and consider the blood oxygen level signal, which can be considered an indicator of neuronal activation in precise neural areas; fMRI revealed that the areas most likely to be activated following a noxious stimulus are the insula, the anterior cingulate cortex, and the somatosensory cortex (primary and secondary). Scalp electroencephalography (EEG) reveals the spontaneous synchronized postsynaptic neuronal activity of the brain cortex. Resting EEG showed the suppression of spontaneous oscillations in healthy volunteers experiencing pain. Finally, the peak alpha frequency recorded in the bilateral temporal scalp has been found to be highly correlated with subjectively rated pain scores, and hence, reflects the pain intensity [5].

The latest definition of “pain” from the IASP (International Association for the Study of Pain), established in 2020, is the following: “An unpleasant sensory and emotional experience associated with, or resembling that associated with, actual or potential tissue damage.” The association adds six features that characterize pain in depth and provide a basis for its assessment:

1. Pain is a personal experience, shaped by biological, psychological, and social factors.
2. Pain and nociception are distinct concepts, with the former being notably more intricate.
3. People develop their understanding of pain throughout their lives.
4. When a patient communicates pain, their expression deserves maximum respect.
5. Despite its protective and adaptive roles, pain can negatively affect psychological and social well-being.
6. Verbal description stands as just one method to portray pain [6].

Physiologically speaking, pain can be described as comprising a sensorial component and an emotional–experiential component. The sensorial component corresponds to the “pure” sensory modality, which enables the reception and transport of potentially harmful stimuli to the central nervous system (CNS). This occurs via a tri-neuronal pathway originating in the periphery and reaching the cerebral cortex. On the other hand, the emotional–experiential aspect, responsible for subjectively assessing the painful impulse, is linked to the limbic system. This complex system of structures is positioned deeply within the telencephalon, mostly near the medial aspect of the cerebral hemispheres, with stimuli reaching here via the thalamus [7]. This projection of painful stimulus to the limbic system is at the heart of the effect of pain on mood, which consists of restlessness and sadness. Conversely, the limbic system also determines the degree of conscientious pain perception: euphoric or in-shock subjects tend not to feel pain, while hypochondriac and anxious subjects feel pain even in response to minimal harmful stimuli. Some studies have shown that listening to music, through its influence on mood, also affects pre-operative anxiety levels and postoperative pain intensity [7,8].

Pain is therefore an experience determined via sensorial and emotional–experiential components, past experiences, psychological structure, and socio-cultural factors [7].

Orofacial pain is a form of pain perceived in the face and/or oral cavity; the term “oral pain” specifically refers to pain perceived in the oral cavity. The first edition of the *International Classification of Orofacial Pain* (ICOP) recognizes seven macro-groups of orofacial pain, based on its etiology and/or localization. They are summarized in Table 1. The most relevant categories in oral medicine include those coded as 1 (including dental, oral mucosal, salivary gland, and jaw bone pains), 2, 3, and 6 (mainly burning mouth syndrome) [9]. Chronic orofacial pain conditions are often associated with behavioral alterations of various natures, ranging from facial expression changes to modifications in everyday life (for example, during food consumption). Behavioral changes often lead to a reduced perception of life quality and an increase in pain intensity [8,10]. The importance of these changes is also demonstrated by the efficacy of cognitive–behavioral treatments in addressing oral chronic pain conditions. When assessing pain behavior (PB), it is helpful to seek an evaluation not only from the patient but also from someone close to them, in order to understand which behaviors can be considered deviations from the normalcy of daily life [3].

A systematic review from 2017 [11] analyzed the existing relationship between pain and depressive syndromes; several clinical studies revealed that chronic pain, as a stressful condition, often induces depression, and that patients with depression due to chronic pain present a worse prognosis when compared to those who do not. Nonetheless, the precise physio-pathological path that justifies these observations still lacks a widely agreed-upon explanation within the scientific community. In any case, anxiety and depression are associated with an increase in perceived oral PI and a reduced QoL perception [12,13].

Different conditions can lead to qualitative and/or quantitative sleep alterations. Acute orofacial pain can cause transitory insomnia that can be resolved by treating the painful condition; orofacial chronic pain, on the other hand, can cause persistent insomnia that tends to develop independently of the painful condition. Some studies have found a bidirectional relationship between sleep disorders and chronic pain, meaning that sleep disorders can also promote chronic pain, probably through mood deterioration, higher stress and disability levels, and lower pain tolerance. Detecting sleep disorders in patients with chronic pain conditions has some therapeutic implications, such as improving sleep hygiene, utilizing cognitive–behavioral therapy, and employing specific pharmacological therapies [14,15].

The aim of this review is to provide an overview of the aspects involved in the orofacial pain phenomenon, especially dental pain, emphasizing the parameters that need to be assessed. Some of the tools used for this purpose will be analyzed, and an effort will be made to determine which of them are to be used in a specific clinical setting.

## 2. Materials and Methods

### 2.1. Focused Questions

What are the critical factors to consider when evaluating orofacial pain, particularly in chronic cases? What are the primary assessment tools used for this purpose?

### 2.2. Eligibility Criteria

Our analysis of studies was guided by the following inclusion criteria: (I) study design—clinical trials, case–control studies, and case reports; (II) participants—individuals with painful conditions and/or psychological implications; (III) interventions—assessment of pain with or without pain-reducing therapies; (IV) outcomes—pain intensity, psychological status, pain assessment tools, and psychological assessment tools. We exclusively considered studies with freely accessible full texts that fulfilled all the inclusion criteria. Additionally, we excluded studies with one of the following features: (I) abstracts of articles published in languages other than English, (II) duplicate studies, (III) in vitro or animal clinical studies, (IV) irrelevant studies, (V) irrelevant articles (namely, reviews and articles whose more recent versions are available), and (VI) studies with no freely accessible full texts.

### 2.3. Search Strategy

To conduct this review, we employed the PICO model (Population, Intervention, Comparison, Outcome) for a systematic literature search using the PubMed (MEDLINE), Scopus, and Web of Science electronic databases (Table S1, Supplementary Materials). We reviewed the abstracts of studies evaluating pain assessment and/or psychological implications.

### 2.4. Research

The medical subject heading (MeSH) terms used were pain assessment, mouth, anxiety, depression, quality of life, behavior, and sleep. An exhaustive electronic search was conducted using the PubMed (MEDLINE), Scopus, and Web of Science databases, targeting articles published between 1983 and 2023. The data extraction process spanned approximately 20 weeks, with the final search conducted on 4 October 2023. Two calibrated reviewers conducted the search, and any disagreements or discrepancies were resolved through consensus or consultation with two additional reviewers. All titles and abstracts from the initial search were thoroughly reviewed, and studies that were not relevant were excluded. Relevant articles were listed and carefully examined for any similar studies meeting our inclusion criteria. The full texts of the included studies were thoroughly read, and their findings were documented.

Details of the search strategies applied to each electronic database are presented in Table S1 (Supplementary Material).

### 2.5. Quality Assessment of Included Studies

To assess the risk of bias in the included studies, we conducted a qualitative analysis of the clinical studies using the National Heart, Lung, and Blood Institute (NHLBI) Quality Assessment Tools for Controlled Intervention Studies, for Observational Cohort and Cross-Sectional Studies.

## 3. Results

**Figure 1:**
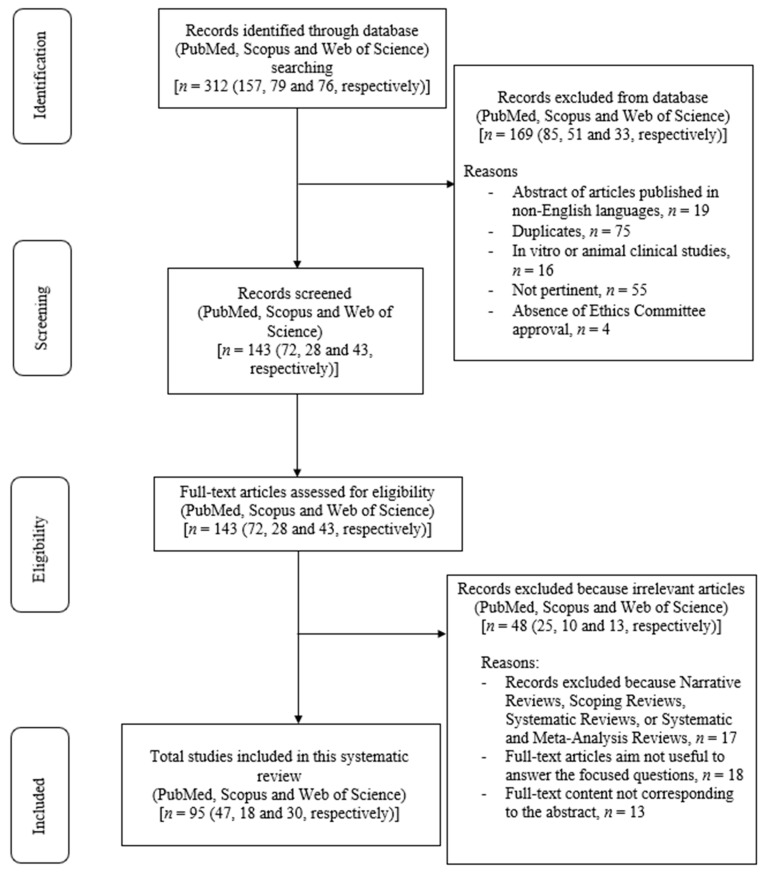
Flowchart of the review process.

The primary search identified 312 articles based on MeSH terms, published from 1983 to 2022. Following this, 169 articles were removed—19 articles with abstracts published in non-English languages, 75 duplicates, 16 in vitro or animal clinical studies, 55 studies that were not pertinent (namely, discussing topics that are unrelated to the ones considered in our review), and 4 with an absence of Ethics Committee approval)—and 143 articles were screened based on their titles and abstracts. The remaining 143 full-text articles were assessed for eligibility. Additionally, 48 articles were excluded because they were irrelevant (reviews, *n* = 17; full-text articles whose aims were not useful to answer our focused questions, *n* = 18; full-text content not corresponding to the abstract, *n* = 13). Finally, 95 relevant articles were included and thoroughly analyzed in this review. The review process is visually represented in Figure 1.

Table S2 (Supplementary Materials) provides details on the studies excluded from this review along with the reasons for their exclusion.

Table S3 (Supplementary Materials) summarizes essential information about the studies included in this review, describing the authors, publication year, study design, number of subjects included, and assessment tools employed.

Table 2 describes the distribution of the references used in this review.

### Risk of Bias

The Cochrane Collaboration tool was applied to assess the risk of bias in the articles included in this review (Table S4, Supplementary Materials), using the judging criteria for risk of bias shown in Table S4 (Supplementary Materials). A moderate risk of bias was observed in this review. Table 3 shows the Risk of Bias of the studies included in this review. All of the literature involved in this study had low risk in the categories of incomplete outcome data and selective reporting.

## 4. Discussion

### 4.1. Pain Intensity Assessment

Pain intensity (PI) is one of the main dimensions that form the sensory component of pain. It is associated with stress, anxiety, and pain coping mechanisms. It is usually the first parameter perceived by the patient, and it also indicates the absence of pain when null. Its assessment is based on different scales that attempt to quantify it using numerical values [16].

The Visual Analogue Scale (VAS) is a PI rating scale made up of a 100 mm straight line, with the threshold values of “no pain” corresponding to a value of 0, and “worst pain” corresponding to a value of 10 (alternatively, 100). Different evidence suggests that VAS is the most used instrument to measure orofacial PI, particularly when assessing it in relation to a specific treatment [17]. In a clinical trial involving more than 1000 patients, an attempt was made to understand the average cutoff of a VAS to distinguish between “moderate pain” and “severe pain”. The results showed that 85% of patients reporting moderate pain chose an average score of 49 mm, while 85% of patients reporting severe pain chose an average score of 75 mm [18]. The VAS demonstrated good reliability and sensitivity, capable of detecting variations in orofacial PI over time. Studies using this scale concluded that the minimum clinically significant difference (MCSD) detectable on a VAS is, on average, 12–13 mm [18,19,20].

**Figure 2:**
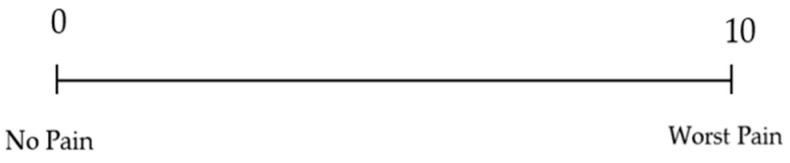
Visual Analogue Scale (VAS).

The main problem associated with this tool is its use in patients such as the elderly, where the abstraction capacities required to define PI as a point on a line may be lacking or absent [21]. By associating VAS with colors or grayscale, the tool can be modified to be easily applied to those patients who find it difficult to use (e.g., those with reduced psychomotor performance); electronic versions of the VAS have been developed [22]. Figure 2 shows a representation of the Visual Analogue Scale [17].

**Figure 3:**
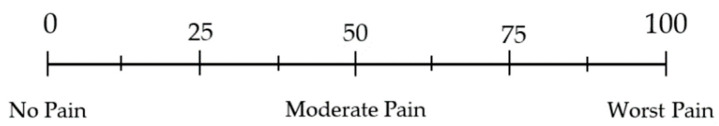
Numerical Rating Scale (NRS).

In the Numerical Rating Scale (NRS), the line on which the patient must place their PI is graduated. This scale has demonstrated a good correlation with other orofacial pain measurement instruments, although it is less “fluid” than the VAS (in this case, not all values between 0 and 100 can be chosen, only those that are graduated). Nevertheless, in some studies, it proved to be more practical to use compared to other assessment tools [23]. A clinical trial conducted in 2001 revealed that on an 11-point NRS, variation in two points (alternatively, 30%) represented the MCSD, especially when assessing changes in PI over time. Just like the VAS, the NRS has been shown to be easily applicable for measuring quantities other than pain, such as nausea. Lastly, the NRS results were more easily applicable in older patients than those of VAS [24]. Figure 3 shows a representation of the Numerical Rating Scale [23].

**Figure 4:**
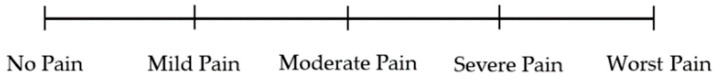
Verbal Rating Scale (VRS).

The Verbal Rating Scale (VRS) uses different adjectives, each associated with a number and arranged in a crescent order, to assess pain; examples of adjectives that can be used include “absent”, “mild”, “moderate”, and “severe”. The VRS also showed a good correlation with other orofacial PI assessment instruments and is applicable in most patients [25]. A strong link has been found between the NRS and VRS, allowing for correspondence to be created between each of the terms used in the VRS and a number in the NRS. The main challenges when using these tools occur when assessing patients who are not native speakers have linguistic difficulties, or whose sensations are not reflected by the adjectives used in the tool [26]. Figure 4 shows a representation of the Verbal Rating Scale [25].

**Figure 5:**
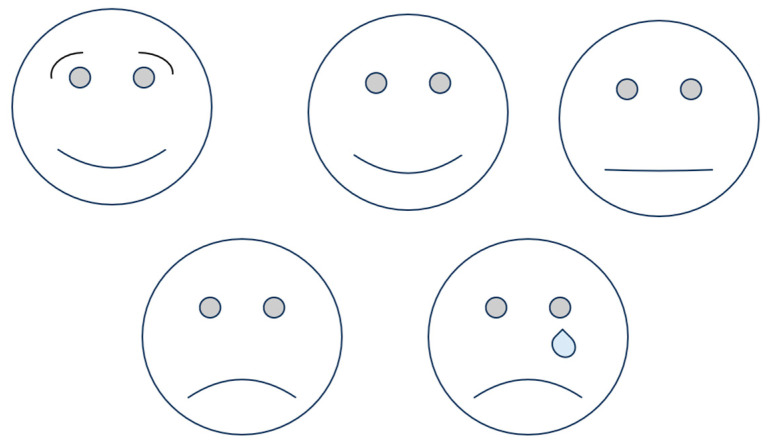
Faces Pain Scale (FPS).

When using the Faces Pain Scale (FPS), the patient is asked to choose, among a series of drawings (or pictures) depicting suffering faces arranged in a crescent order, the facial expression that best represents their pain [27]. This scale is currently used to assess orofacial (especially dental) PI in elderly patients, in which the tool has demonstrated good validity and reliability in measuring chronic and acute pain; both the classic version (Wong-Baker) and the revised one (FPS-R) have been shown to be equally valid. Finally, electronic versions of the FPS have been developed and, despite yielding slightly higher values of PI compared to the classic FPS, still fall within acceptable limits [28,29,30,31]. Figure 5 shows a representation of the Faces Pain Scale [27].

The Relief Assessment Scale (RAS) is used to assess relief from a painful condition; it can be graphically represented in an analogous way to the VAS, NRS, or VRS. Although statistically significant differences among the different representations did not emerge, the verbal form seems to be the most appropriate for describing relief. Even though in a brief time frame (≤24 h), relief assessment has proven to be valid and reliable, in a longer time frame, it appears to be more related to mood and psychological distress rather than effective PI reduction [32,33].

The VAS, NRS, VRS, and FPS-R are the most used tools for orofacial PI assessment. A clinical study [34] compared the ability of the four tools to measure PI following thermic stimulation; the results showed little difference among the scales, with the four tools arranged in a crescent order of responsivity as follows: NRS, VAS, VRS, and FPS-R. A similar study [35] also demonstrated that the four scales are strongly correlated with one another; furthermore, it showed that while the VAS and NRS tend to be less influenced by non-PI-related factors, the VRS also reflects pain interference, and the FPS-R also reflects pain unpleasantness. Lastly, the NRS was found to be the most sensitive instrument when used to assess PI in patients with physical disabilities [36].

Pain Assessment in Advanced Dementia (PAINAD) is a scale used in patients with severe cognitive impairment and non-compliant patients. Due to the communicative disability of these patients, PI assessment is based on the observation of five indicators (breathing, vocalization, facial expression, body language, and consolability), each of which is assigned a score that allows for compatibility with numerical scales. Its usefulness has been demonstrated by some studies that revealed that PI self-reporting in cognitively impaired patients is insufficient [37]. Despite PAINAD being easily administered to the patients for which it was created, several studies have shown its reduced ability to assess PI variations following therapy [38,39].

When assessing dental-related acute pain conditions (such as those characterized by apical periodontitis, an abscess, or pulp inflammation), the unidimensional tools VAS, VRS, and NRS have proven to be the most suitable [40,41,42,43]. A study conducted on twenty-five adult patients suffering from dentine hypersensitivity used the VAS and the NRS (along with other verbal tools) to assess their discomfort due to the condition, confirming previous results indicating that both verbal and non-verbal scales are useful for quantifying the sensory aspect of pain [42].

#### Pain Intensity Assessment in Children

When assessing PI in children, particular attention must be given to the tool used, in order to have a more realistic description of the pain. The VAS is efficiently applicable to children older than 7 years old, being able to represent PI in a valid and reliable manner [22]. When using the VRS, particular care must be taken, since some children may have problems with understanding the real meaning of the adjective used [26].

The main challenges when using these tools occur when assessing patients who are not native speakers, have linguistic difficulties, or whose sensations are not reflected by the adjectives used in the tool [26].

The FPS is the most commonly used tool to assess PI in children, who tend to prefer graphic representations to describe their sensations [28,29,30]. It is also currently used to assess orofacial (especially dental) PI in child patients, having demonstrated good validity and reliability in measuring both chronic and acute orofacial pain conditions. Additionally, the FPS has been shown to be a reliable and valid instrument to assess acute dental pain, sometimes being more sensitive than the VAS when used in pediatric patients [44].

Self-report information on oral pain is not obtainable in some patients, such as preverbal children. To assess the presence of oral pain in these categories, the Dental Discomfort Questionnaire (DDQ) has been created. The parent or the caregiver is asked about the child’s toothache, chewing habits, crying, or earache episodes during meals, etc.; for each question, there are three possible answers: “never”, “sometimes”, and “always”, each one with a corresponding score. The questionnaire showed enough specificity and sensitivity to be used as a predictor of the existence of toothache in preverbal children [45]. A study from 2020 found an association between higher DDQ scores and socioeconomic variables such as a non-nuclear family structure, lower monthly income, parents with a lower level of education, and a poor perception of the child’s oral health [46]. Other studies correlated higher scores with dental treatment needs and more invasive procedures [47]. A study from 2021 attempted to use the DDQ to assess oral pain in children with cognitive impairment. When compared to cognitively normal children, the answers given by the test group were statistically similar, showing the tool to be a descriptive, functional, and easy-to-use questionnaire for children with intellectual disabilities in the assessment of oral pain intensity [48].

### 4.2. Pain Localization Assessment

Pain localization (PL) assessment is important because it permits the targeting of global pain assessment to a specific anatomic district and allows for monitoring of the therapeutic response more efficiently. Having medical staff understand the PL more deeply increases patients’ trust in them [49,50].

Pain maps are graphic representations of the human body where the patient is asked to mark the areas where they feel pain; they can eventually associate different colors with different PIs. This technique is particularly useful in children and non-verbal patients. Despite some studies showing that pain maps are less suitable for describing PI (such as the VAS and NRS), they help a lot in the identification of PL and pain distribution, being able to influence the therapeutic approach, especially when discussing the topic [49]. Pain maps have been employed for different purposes, such as pelvic and knee pain assessment [50,51].

When assessing oral pain localization in BMS, the most commonly referenced sites are the tongue, gums, lips, and palate [52]. Up to two-thirds of patients report a burning sensation on the tongue [53].

### 4.3. Pain Quality Assessment

Multidimensional tools permit a much more comprehensive pain assessment that includes not only PI, but also psychological, emotional, and behavioral factors. Methods like the VAS and NRS can be used, but some problems have been encountered with these approaches when assessing and discriminating conditions where the borderline is much thinner, as well as distinguishing the sensorial and emotional–experiential components [54]. More complex instruments have then been developed, usually in the form of questionnaires, in which the different questions (called “items”) are classified into classes and subclasses that represent the different life aspects that can be influenced by the pain experience. When administering questionnaires, translations, semantic correspondence, and medical staff’s relational abilities have a great influence [55].

The McGill Pain Questionnaire (MPQ) permits the assessment of the PI (usually through a VAS), PL (through a pain map), sensorial aspects (intensity, localization, temporality, and quality), and affective aspects (quality and behavior—e.g., alleviating and aggravating factors) of orofacial pain. The adjectives that are used showed statistically significant differences from one another, following which classification into eight classes was possible [54]. The scores that can be calculated when the test has been completed are [56]:

- The Pain Rating Index (PRI), which refers to PI, globally and for each class.
- Constellation Words, which are the words commonly used to describe pain related to a specific condition. The adjectives belonging to this definition are usually the ones used by at least 33% of patients who have that condition. The most used words for describing pain in burning mouth syndrome (BMS) are, for example, “hot/burning”, “radiating”, “tiring/exhausting”, and “fearful” [57].
- The Number of Words Chosen (NWC), which corresponds to the total number of words chosen, with the frequency rate of each.

The MPQ is also efficiently used to verify different approaches’ effectiveness, particularly when assessing low-back pain [56,57]. This tool has been modified to create a shorter version (SF-MPQ), which is easier to apply in every clinical setting. The validity and reliability of this questionnaire have also been demonstrated [58].

The assessment of dental pain quality may aim to verify pulp vitality (therefore, it should be considered a vitality assessment, not a pain assessment), consisting of thermal and electrical stimulation. Percussion and/or palpation tests help in the research of periodontal and root involvement [59].

### 4.4. Pain Behavior Assessment

Pain behavior (PB) assessment is usually associated with multidimensional questionnaire administration and can be carried out through the completion, by the patient, of daily activities and a pain diary. Using this instrument, the patient indicates the daily moments when they perform specific actions (such as sitting, lying down, eating, sleeping, practicing sexual activities, taking drugs, and engaging in leisure activities), eventually determining the time dedicated to each of them; with the same diary (or alternatively, in a different one), the patient also has to indicate the moments when they feel oral pain (eventually determining the PI). The main advantage of using a daily diary is being able to detect a correlation between specific activities and the orofacial pain condition, assessing the disease trend, and avoiding mistakes when asking the patient to describe past sensations [60,61,62]. For the diary to be a valid assessment tool, sufficient adhesion and constancy are required during completion [61]. A study in 2019 tried to evaluate the use of a comfort diary instead of a pain diary, resulting in completely different (and not opposite) results; this indicates that pain and relaxation belong to different nervous areas [63]. PB assessment can also be carried out by observing patients while they are moving and talking, looking for behavior that can indicate the sensation of pain, such as a sharp contortion of the face, brow lowering, lid tightening, cheek rising, mouth stretching, eye closing, and lid shutting. Other signs include low and soft indistinguishable sounds; stiff, tensed muscles of the extremities; and the act of forming a fist [64]. The Behavioral Pain Scale (BPS) received validation to be applied in non-communicative adult patients; the main parameters analyzed are facial expression, hip movement, and compliance with ventilation. This instrument is proven to be one of the instruments with the strongest psychometric properties, having been validated in multiple countries and in multiple languages [65,66]. The Pain Sensitivity Questionnaire (PSQ) consists of asking the patient what, in their opinion, the PI would be in different contexts (for example, when burning their tongue, after physical training, or after a sunburn on the shoulders). Thereby, it is possible to give an oral PI and PB assessment. The PSQ has been validated in different languages, including English, French, and Norwegian [67,68,69].

### 4.5. Quality of Life Assessment

Chronic pain-affected patients’ quality of life (QoL) is usually reduced, both due to the suffering deriving from pain (sensorial and emotional) and functional and social limitations. There is a bidirectional relationship between QoL and pain, following which pain is a predictor of low QoL, sadness, depression, and anger; a reduced perception of QoL, in addition, is a predictor of future pain [70,71].

The Short-Form 36 (SF-36) assesses life aspects such as physical functioning, role limitations due to health or emotional problems, energy levels, emotional well-being, social functioning, pain, and general health status. It has proven to be a valid and reliable tool for assessing QoL, being easy to complete, and well-accepted by patients [72]. A twelve-item version of the questionnaire has been developed (SF-12) and, despite a higher percentage of errors, it has been able to efficiently reflect the results of the 36-item version [73].

QoL assessment is also possible for specific issues, as seen in the Oral Health Impact Profile (OHIP), which assesses the impact of oral health on QoL. It considers functional limitations (speech and taste), physical pain (at rest and when eating), psychological discomfort (self-consciousness and tension), physical disability (unsatisfactory diet and meal interruption), psychological disability (difficulties in relaxing and embarrassment), social disability (irritability with other people and difficulties in daily tasks), and handicap (reduced life satisfaction and total inability to function). The OHIP has shown validity in assessing QoL in patients with dental problems [74]. Recently, a shorter version composed of twelve instead of fourteen items, and with three instead of four possible answers to each item, resulted in a psychometrically improved version of the instrument [75]. The usage of OHIP in assessing oral health-related QoL is valid for different conditions, such as xerostomia, temporomandibular disorders, pregnancy, and BMS [76,77,78].

Patients with BMS showed higher OHIP-14 scores when compared to controls, with particular attention paid to the parameters “feeling tense”, “difficulty in relaxing”, “irritability”, “difficulty in working”, and “finding life less satisfying” [52]. OHIP-14 has been compared to the General Oral Health Assessment Index (GOHAI) in assessing BMS patients, resulting in a better tool for evaluating this condition [79]. Lastly, the SF-36 questionnaire has been proven to be a valid instrument to assess BMS patients, as indicated by the higher scores in these patients when compared to controls [80].

### 4.6. Psychological Assessment

The general mechanisms through which the pain and psychological components of the individual influence each other are indicated in the Introduction. Psychological involvement also has important implications for therapies and justifies the usefulness of cognitive–behavioral therapy.

#### 4.6.1. Anxiety and Depression Assessment

The Hamilton Anxiety Rating Scale (HAM-A) and the Hamilton Depression Rating Scale (HAM-D) assess anxiety and depression, respectively. They consider parameters such as anxious mood, tension, fears, insomnia, intellectual alterations, suicidal thoughts, agitation, and somatic symptoms correlated with these conditions. They have been proven valid and reliable in various clinical contexts, ranging from the psychological to rheumatological fields (as in vulvodynia and BMS) [81,82,83].

The State–Trait Anxiety Inventory (STAI) is used to specifically assess anxiety, distinguishing between trait anxiety (which is associated with a stable and persistent mood) and state anxiety (which is referred to a specific moment or context). It has been shown to be a valid instrument for assessing both psychological and physical conditions [84,85]. This tool permits the differentiation of anxiety from other symptoms or conditions affecting patients, eliminating confounding factors and analyzing specifically the effect of anxiety on the individual [86].

The Beck Depression Inventory (BDI) specifically addresses depression, particularly in chronic pain conditions. It has been proven to be a valid and reliable tool in several studies, serving as a screening instrument to guide further detailed assessments [87]. Lastly, it is a valid, relatively short, and easily administered instrument for chronic spinal pain [88].

Another tool is the Hospital Anxiety and Depression Scale (HADS), which assesses anxiety and depression both in combination (HADS-T) and separately (HADS-A and HADS-D subscales). The items considered include tension, irritability, loss of interest, fears, optimism, alarming thoughts, mood, relaxation, slowness in movement, and other parameters. It is a valid instrument for the preliminary assessment of anxiety and depression in patients in different contexts, ranging from rheumatological (as in axial spondyloarthritis) to tumoral contexts [89,90]. The feature of assessing anxiety and depression together makes this instrument, on the one hand, more suitable for administration by non-psychologically specialized personnel, and on the other hand, less suitable for a detailed evaluation; consequently, the HADS can be considered a useful screening tool [91].

All these components are fundamental in assessing patients with chronic oral pain conditions, such as BMS. When these patients are assessed using the instruments shown in this paragraph, higher anxiety levels, both for the state and trait forms, and higher depression levels are often observed [52,92]. A 2012 study found that among the 100 postmenopausal elderly women with BMS analyzed, only 20% of them did not show any signs of anxiety or depression [93].

#### 4.6.2. Pain Coping Mechanism Assessment

Other relevant psychological factors concerning pain include coping mechanisms, pain acceptance, and tolerance. Facing pain in a passive manner, for example, has been shown to be a predictor for disabling back and neck pain in patients affected by pain in those areas. Some studies have revealed that personality disorders can arise as consequences of chronic pain conditions. On the other hand, other studies have revealed that certain personality features, especially emotional instability, can promote the onset of chronic pain conditions [94].

The Ten-Item Personality Inventory (TIPI) is a questionnaire developed to assess five personality aspects in a subject, specifically, (1) extraversion, (2) agreeableness, (3) conscientiousness, (4) emotional stability, and (5) openness to experience. Each aspect is assessed by considering two of the ten questions in the TIPI. This questionnaire has been found to be valid in different languages, including Chinese, Norwegian, and Portuguese [95,96,97]. When used in patients with BMS, the TIPI revealed that patients presenting a psychological component are characterized by greater emotional instability, reduced openness to experience, and reduced extraversion compared to patients without a psychological component. Emotional instability, moreover, is associated with oral PI and the severity of the clinical condition [98].

The Pain Catastrophizing Scale (PCS) is a thirteen-item tool that assesses the patient’s fear of pain, often resulting in the belief that facing the pain is impossible. It has been validated as an instrument for chronic pain patients, both in the form of a questionnaire and in the form of a daily diary [99,100]. Pain catastrophizing is a common feature of patients with BMS, indicating that treatment approaches targeting catastrophizing, pain self-efficacy, and acceptance may prove beneficial in improving mood and oral-related quality of life in these patients [79,101].

### 4.7. Sleep Disorder Assessment

The two most widely used tools for sleep assessment are the Epworth Sleepiness Scale (ESS) and the Pittsburgh Sleep Quality Index (PSQI). The ESS assesses the probability of the individual falling asleep in different contexts, whether daily or not. The PSQI assesses sleep quality over the last month by considering the patient’s answers, as well as those of individuals who sleep near them. The items considered in the PSQI include subjective quality, duration, efficacy, sleep disturbances during the night and day, and the use of sleeping medication. The ESS has been validated and proven reliable for assessing diurnal sleepiness [102,103], except for elderly patients, who in some studies, were unable to reliably complete the questionnaire [104]. Regardless of the type of patient it is administered to, the ESS was found to be more valid and reliable when administered by medical professionals [105]. The PSQI is a valid and reliable instrument for assessing the overall quality of sleep and is applicable in both clinical and research contexts [86]. It is particularly useful when assessing insomnia due to chronic pain, both for diagnosis and for evaluating therapy efficiency [106,107].

A study conducted in 2020 on postmenopausal women reporting sleep disorders showed that this condition can lead to the worsening of oral symptoms in BMS. Indeed, pain intensity (PI) in BMS patients was significantly higher in the sleep disorders group than in the control group, and the severity of the complaints was correlated with the presence of depression, anxiety, hostility, phobias, and psychosis [108]. Another study found that BMS patients also had higher scores in the PSQI, correlated with pain interference during daily life [109]. Lastly, a study with 140 patients (70 with BMS and 70 controls) found that sleep disturbances were present in 67.1% of patients in the test group and only in 17.1% in the control group. It also found that PSQI scores were positively correlated with EES, VAS, HAD-A, and HAD-D scores [110].

### 4.8. Global Pain Assessment

The different aspects comprising the experience of orofacial pain have been shown, each one using some of these assessment tools. Table 4 summarizes all these concepts. Using one tool over the others in a single patient requires evaluating their condition, cognitive capacity, and language knowledge.

### 4.9. Research Limitations and Future Research

The main limitations associated with this review are related to the characteristics of pain itself: pain, particularly when chronic, is a subjective experience, whose features differ among patients in relation to etiology, the patient’s personality, and their coping mechanisms. This variability makes it hard to assess pain in a detached and objective way and to compare the results obtained in different studies.

Future research on pain assessment should focus on particular categories of patients (such as pediatric, elderly, mentally ill, and chronically ill) and on instrumental assessment tools (such as functional magnetic resonance imaging). This measurement method could also help to overcome the problem related to the subjectivity of the pain experience.

## 5. Conclusions

Understanding pain in its multifaceted dimensions is pivotal for tailoring effective treatments and enhancing patient care. Pain intensity assessment, often the initial parameter, is crucial for evaluating its impact and progression. Tools like the Visual Analogue Scale (VAS), Numerical Rating Scale (NRS), Verbal Rating Scale (VRS), and Faces Pain Scale (FPS) offer valuable insights into pain levels and aid in treatment monitoring. Pain localization assessment through techniques like pain maps provides a targeted approach, facilitating precise pain management. Exploring pain quality and behavioral manifestations enriches our understanding. Tools like the McGill Pain Questionnaire (MPQ) and Pain Behavior Scale (PBS) unravel the pain perception intricacies, aiding in tailored interventions. Evaluating coping mechanisms and personality traits sheds light on individual pain responses, and is valuable for personalized pain management plans.

Chronic orofacial pain conditions profoundly affect quality of life, necessitating comprehensive evaluation through instruments like Short-Form 36 (SF-36) and the Oral Health Impact Profile (OHIP). Additionally, exploring psychological factors like anxiety and depression underscores the importance of mental health in pain assessment and management. Tools like the Hamilton Anxiety and Depression Rating Scales offer insights into pain’s emotional aspect, promoting integrated care approaches. Incorporating sleep assessment into pain evaluation is crucial, given the reciprocal relationship. The Epworth Sleepiness Scale (ESS) and Pittsburgh Sleep Quality Index (PSQI) provide comprehensive insights into sleep patterns and their association with pain, contributing to a comprehensive understanding of a patient’s condition.

In conclusion, a thorough and multidimensional assessment of orofacial pain is vital for effective pain management and improved patient outcomes. By integrating various assessment tools and considering the holistic aspects of pain, clinicians can tailor treatments to individual needs, alleviate suffering, and ultimately enhance the lives of those enduring oral and dental pain.

## Figures and Tables

**Table 1:** First edition of the *International Classification of Orofacial Pain* (ICOP).

Code	Classes
1	Orofacial pain attributed to disorders of dentoalveolar and anatomically related structures
2	Myofascial orofacial pain
3	Temporomandibular joint pain
4	Orofacial pain attributed to lesion or disease of the cranial nerves
5	Orofacial pains resembling presentations of primary headaches
6	Idiopathic orofacial pain
7	Psychosocial assessment of patients with orofacial pain

**Table 2:** Structure of the references in this review.

	Topic	References
4.1	Pain Intensity Assessment	[16,17,18,19,20,21,22,23,24,25,26,27,28,29,30,31,32,33,34,35,36,37,38,39,40,41,42,43,44,45,46,47,48]
4.2	Pain Localization Assessment	[49,50,51,52,53]
4.3	Pain Quality Assessment	[54,55,56,57,58,59]
4.4	Pain Behavior Assessment	[60,61,62,63,64,65,66,67,68,69]
4.5	Quality of Life Assessment	[70,71,72,73,74,75,76,77,78,79,80]
4.6	Psychological Assessment	[81,82,83,84,85,86,87,88,89,90,91,92,93,94,95,96,97,98,99,100,101]
4.7	Sleep Disorder Assessment	[102,103,104,105,106,107,108,109,110]

**Table 3:** Risk of bias of the studies included in this review: the green symbol represents a low risk of bias, while the yellow symbol represents a high risk of bias.

	Random Sequence Generation	Allocation Concealement	Blinding
Treister et al., 2019 [16]	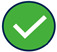	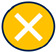	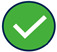
Shafshak et al., 2021 [17]	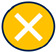	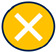	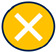
Kendrick et al., 2005 [18]	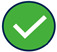	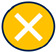	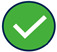
Taddio et al., 2009 [19]	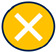	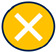	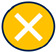
Todd et al., 2017 [20]	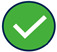	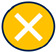	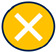
Closs et al., 2004 [21]	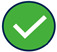	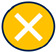	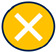
Lewinson et al., 2013 [22]	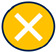	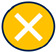	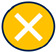
Ruskin et al., 2014 [23]	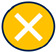	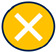	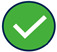
Wikstrom et al., 2018 [24]	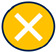	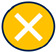	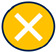
Alghadir et al., 2018 [25]	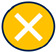	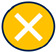	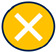
Jenkins et al., 2009 [26]	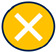	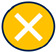	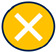
Hicks et al., 2001 [27]	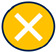	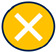	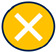
Suraseranivongse et al., 2005 [28]	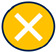	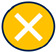	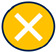
Sun et al., 2015 [29]	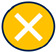	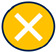	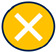
Gulur et al., 2009 [30]	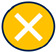	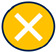	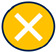
Fadayevatan et al., 2019 [31]	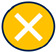	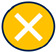	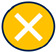
Lee et al., 2015 [32]	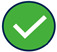	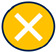	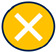
Girandeau et al., 2004 [33]	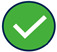	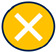	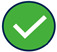
Ferreira-Valente et al., 2011 [34]	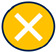	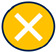	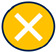
Thong et al., 2018 [35]	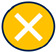	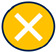	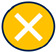
Miró et al., 2016 [36]	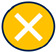	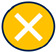	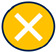
Malara et al., 2016 [37]	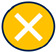	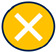	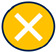
Ersek et al., 2010 [38]	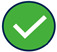	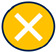	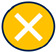
Paulson-Conger et al., 2011 [39]	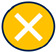	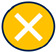	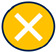
De-Figuerido et al., 2020 [40]	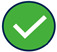	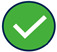	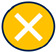
Tran et al., 2023 [41]	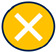	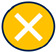	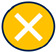
Odai et al., 2015 [42]	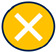	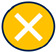	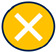
Shah et al., 2012 [43]	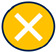	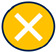	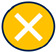
Khatri et al., 2012 [44]	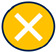	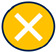	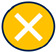
Versloot et al., 2004 [45]	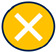	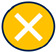	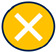
Felipak et al., 2020 [46]	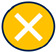	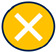	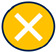
Daher et al., 2015 [47]	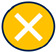	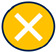	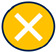
Senirkentli et al., 2021 [48]	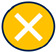	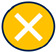	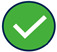
Mendonça et al., 2018 [49]	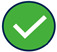	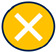	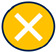
Aibel et al., 2023 [50]	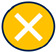	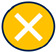	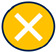
Elson et al., 2011 [51]	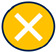	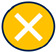	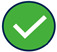
Adamo et al., 2020 [52]	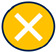	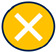	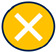
Sevrain et al., 2015 [53]	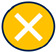	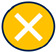	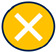
Melzack et al., 1985 [54]	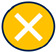	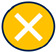	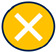
Kachooei et al., 2015 [55]	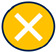	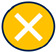	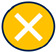
Fontana Carvalho et al., 2020 [56]	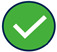	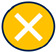	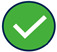
Renovato França et al., 2010 [57]	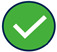	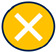	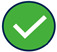
Dworkin et al., 2015 [58]	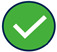	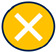	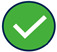
Erdogan et al., 2019 [59]	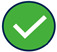	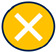	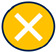
Lewandowski et al., 2009 [60]	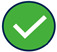	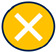	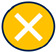
Vertsberger et al., 2022 [61]	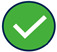	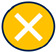	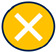
Karoly et al., 2014 [62]	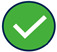	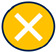	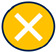
Gruszka et al., 2019 [63]	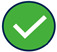	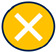	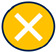
Mitra et al., 2020 [64]	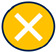	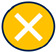	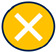
Delgado et al., 2021 [65]	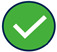	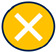	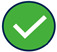
Gomarverdi et al., 2019 [66]	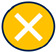	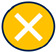	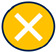
Ruscheweyh et al., 2012 [67]	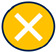	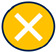	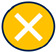
Sellers et al., 2020 [68]	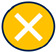	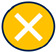	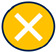
Bell et al., 2018 [69]	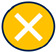	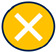	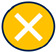
Heary et al., 2022 [70]	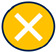	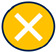	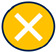
Müller et al., 2017 [71]	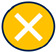	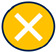	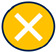
Kwan et al., 2016 [72]	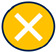	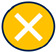	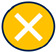
Kishi et al., 2005 [73]	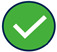	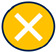	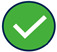
Campos et al., 2021 [74]	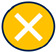	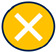	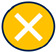
Omara et al., 2021 [75]	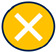	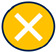	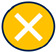
Musskopf et al., 2018 [76]	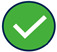	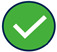	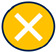
Yule et al., 2015 [77]	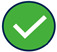	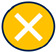	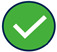
Serrano et al., 2022 [78]	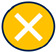	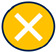	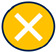
Chana et al., 2021 [79]	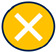	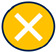	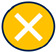
López-Jornet et al., 2008 [80]	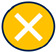	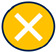	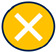
Kyle et al., 2016 [81]	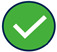	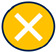	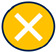
Munhoz Carneiro et al., 2015 [82]	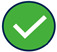	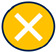	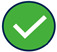
Meltzer-Brody et al., 2009 [83]	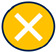	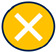	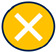
Donham et al., 1984 [84]	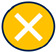	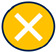	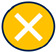
Canfora et al., 2022 [85]	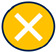	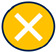	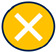
Zitser et al., 2022 [86]	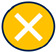	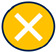	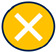
Freedland et al., 2019 [87]	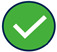	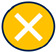	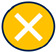
Choi et al., 2014 [88]	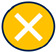	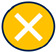	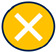
Chan et al., 2017 [89]	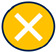	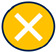	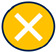
Nipp et al., 2018 [90]	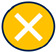	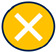	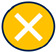
Silva et al., 2017 [91]	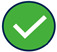	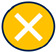	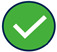
Sikora et al., 2018 [92]	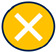	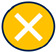	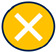
Malik et al., 2012 [93]	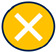	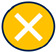	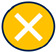
Burns et al., 2012 [94]	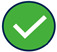	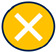	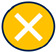
Shi et al., 2022 [95]	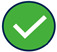	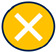	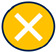
Nunes et al., 2018 [96]	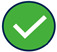	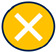	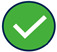
Thørrisen et al., 2021 [97]	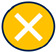	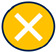	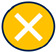
Huyan et al., 2021 [98]	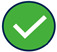	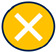	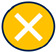
Darnall et al., 2017 [99]	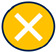	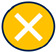	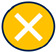
Cano et al., 2005 [100]	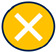	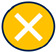	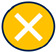
Roguli et al., 2014 [101]	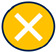	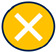	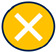
Walker et al., 2020 [102]	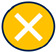	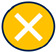	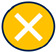
Sap-Anan et al., 2021 [103]	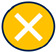	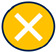	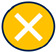
Frohnhofen et al., 2009 [104]	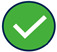	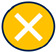	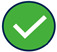
Damiani et al., 2013 [105]	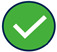	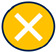	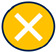
Adamo et al., 2018 [106]	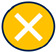	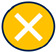	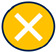
Kirmizigil et al., 2020 [107]	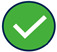	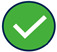	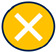
Lee et al., 2020 [108]	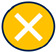	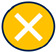	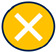
Lee et al., 2022 [109]	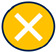	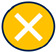	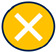
López-Jornet et al., 2014 [110]	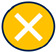	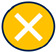	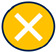

**Table 4:** Global pain assessment.

Aspect	Meaning	Assessment Tools
Intensity	Related to stress, anxiety, and absent pain coping mechanisms	VAS, NRS, VRS, FPS, RAS, PAINAD, DDQ
Localization	Allows for a targeted pain assessment and therapeutic response	Pain maps, MPQ
Quality	Permits doctors to distinguish the sensorial and the emotional–experiential components of pain	MPQ
Behavioral implications	Related to low QoL perception and PI increase	Clinical evaluation, pain diary, BPS, PSQ
Quality of life	Predictor of future pain	SF-36, OHIP
Psychological aspects	Related to perceived PI increase and low QoL perception	HAM-A, HAM-D, STAI, BDI, HADS, TIPI, PCS
Sleep disturbances	Related to mood worsening, higher stress and disability, and pain tolerance lowering	ESS, PSQI

## Data Availability

Data supporting the reported results are available on request from the corresponding authors.

## Supplementary Materials

The following supporting information can be downloaded at: https://www.mdpi.com/article/10.3390/dj11110246/s1, Table S1: Search Strategies for electronic databases; Table S2: Summary table of studies excluded in this comprehensive review; Table S3: Summary table of studies included in this comprehensive review; Table S4: Criteria for judging risk of bias in the “Risk of bias” assessment tool.
