# Supplementary material for: Pain Assessment in Oral Medicine through Its Different Dimensions: A Comprehensive Review
Source: Dent J (Basel). 2023 Oct 24;11(11):246. doi: 10.3390/dj11110246 (PMC10670171; doi:10.3390/dj11110246)
Supplement: Supplementary file 1 [file dentistry-11-00246-s001.zip › dentistry-2585444-supplementary.pdf]

Table S1. Search Strategies for electronic databases.

Database	Search strategy
PubMed (MEDLINE)	#1 "Pain Measurement" [MESH] OR (Measurement, Pain) OR (Pain Measurements) OR (Pain Assessment) OR (Assessment, Pain) OR (Pain Assessments)
	#2 "Mouth" [MESH] OR (Oral Cavity) OR (Cavity, Oral) OR (Oral Cavity Proper) OR (Mouth Cavity Proper) OR (Cavitas oris propria)
	#3 "Anxiety" [MESH] OR "Angst" OR "Hypervigilance" OR "Nervousness" OR "Anxiousness"
	#4 "Depression" [MESH] OR "Depressive Symptoms" OR "Depressive Symptom" OR "Symptom, Depressive"
	#5 "Quality of Life" [MESH] OR "Life Quality" OR "Health-Related Quality Of Life" OR "Health Related Quality Of Life" OR "HRQOL"
	#6 "Behavior" [MESH] OR "Behaviors" OR "Acceptance Processes" OR "Acceptance Process" OR "Process, Acceptance", OR "Processes, Acceptance"
	#7 "Sleep" [MESH] OR "Sleeping Habits" OR "Sleep Habits" OE "Habit, Sleep" OR "Habits, Sleep" OR "Sleep Habit" OR "Sleeping Habit"
	#8 #1 AND #2 AND #3
	#9 #1 AND #2 AND #4
	#10 #1 AND #2 AND #3 AND #4
	#11 #1 AND #2 AND #5
	#12 #1 AND #2 AND #6

	#13 #1 AND #2 AND #7
SCOPUS	#1 “Pain Measurement” [MESH] OR (Measurement, Pain) OR (Pain Measurements) OR (Pain Assessment) OR (Assessment, Pain) OR (Pain Assessments)<br><br>#2 “Mouth” [MESH] OR (Oral Cavity) OR (Cavity, Oral) OR (Oral Cavity Proper) OR (Mouth Cavity Proper) OR (Cavitas oris propria)<br><br>#3 “Anxiety” [MESH] OR “Angst” OR “Hypervigilance” OR “Nervousness” OR “Anxiousness”<br><br>#4 “Depression” [MESH] OR “Depressive Symptoms” OR “Depressive Symptom” OR “Symptom, Depressive”<br><br>#5 “Quality of Life” [MESH] OR “Life Quality” OR “Health-Related Quality Of Life” OR “Health Related Quality Of Life” OR “HRQOL”<br><br>#6 “Behavior” [MESH] OR “Behaviors” OR “Acceptance Processes” OR “Acceptance Process” OR “Process, Acceptance”, OR “Processes, Acceptance”<br><br>#7 “Sleep” [MESH] OR “Sleeping Habits” OR “Sleep Habits” OE “Habit, Sleep” OR “Habits, Sleep” OR “Sleep Habit” OR “Sleeping Habit”<br><br>#8 #1 AND #2 AND #3<br><br>#9 #1 AND #2 AND #4<br><br>#10 #1 AND #2 AND #3 AND #4<br><br>#11 #1 AND #2 AND #5<br><br>#12 #1 AND #2 AND #6<br><br>#13 #1 AND #2 AND #7

Web of Science	#1 “Pain Measurement” [MESH] OR (Measurement, Pain) OR (Pain Measurements) OR (Pain Assessment) OR (Assessment, Pain) OR (Pain Assessments)<br><br>#2 “Mouth” [MESH] OR (Oral Cavity) OR (Cavity, Oral) OR (Oral Cavity Proper) OR (Mouth Cavity Proper) OR (Cavitas oris propria)<br><br>#3 “Anxiety” [MESH] OR “Angst” OR “Hypervigilance” OR “Nervousness” OR “Anxiousness”<br><br>#4 “Depression” [MESH] OR “Depressive Symptoms” OR “Depressive Symptom” OR “Symptom, Depressive”<br><br>#5 “Quality of Life” [MESH] OR “Life Quality” OR “Health-Related Quality Of Life” OR “Health Related Quality Of Life” OR “HRQOL”<br><br>#6 “Behavior” [MESH] OR “Behaviors” OR “Acceptance Processes” OR “Acceptance Process” OR “Process, Acceptance”, OR “Processes, Acceptance”<br><br>#7 “Sleep” [MESH] OR “Sleeping Habits” OR “Sleep Habits” OE “Habit, Sleep” OR “Habits, Sleep” OR “Sleep Habit” OR “Sleeping Habit”<br><br>#8 #1 AND #2 AND #3<br><br>#9 #1 AND #2 AND #4<br><br>#10 #1 AND #2 AND #3 AND #4<br><br>#11 #1 AND #2 AND #5<br><br>#12 #1 AND #2 AND #6<br><br>#13 #1 AND #2 AND #7

Table S2. Summary table of studies excluded in this comprehensive review

Excluded Studies	Exclusion Reasons
Menendez et al., 2016 **[1]**	Comprehensive Review
Hawker et al., 2011 **[2]**	Comprehensive Review
Hajihasani et al., 2019 **[3]**	Systematic Review
Khalid et al., 2012 **[4]**	Comprehensive Review
Xu et al., 2020 **[5]**	Comprehensive Review
Raja et al., 2020 **[6]**	Comprehensive Review
Lindsay et al., 2021 **[7]**	Comprehensive Review
Gorczyca et al., 2013 **[8]**	Comprehensive Review
Pigg et al., 2020 **[9]**	Comprehensive Review
Lin et al., 2022 **[10]**	Comprehensive Review
Sheng et al., 2017 **[11]**	Comprehensive Review
Michealides et al., 2019 **[12]**	Comprehensive Review
Rogers et al., 2022 **[13]**	Meta-analysis
Finan et al., 2014 **[14]**	Comprehensive Review

Whibley et al., 2019 **[15]**	Systematic Review

Table S3. Summary table of studies included in this comprehensive review

Authors and Year of Pubblication	Study Design	Assessment tool(s)	Subjects	Type of pain
Treister et al., 2019 **[16]**	Randomized Controlled Trial	VAS<br>MPQ<br>NPS<br>PD-Q<br>BAI<br>BDI<br>EIHS<br>FMRIB	68	Sub-acute back pain (SBP)<br>Chronic Low-back pain (CLBP)
Shafshak et al., 2021 **[17]**	Cross-sectional Study	VAS<br>NRS	100	CLBP
Kendrick et al., 2005 **[18]**	Prospective Descriptive Trial Study	NRS	354	Acute pain (general)
Taddio et al., 2009 **[19]**	Randomized Controlled Trial	VAS	120	Due to immunization injections
Todd et al., 2017 **[20]**	Randomized Controlled Trial	VAS	48	Acute pain due to trauma
Closs et al., 2004 **[21]**	Comparative Study	VRS<br>NRS<br>FPS<br>CAS<br>MVAS	113	Acute and chronic pain (general)
Lewinson et al., 2013 **[22]**	Descriptive Laboratory Study	CVAS	36	Due to pathellofemoral pain syndrome
Ruskin et al., 2014 **[23]**	Cross-sectional Study	NRS<br>CAS	143	Chronic pain (general)
Wikström et al., 2018 **[24]**	Repeted Measurement Study	NRS<br>VRS	479	Nausea

Alghadir et al., 2018 **[25]**	Repeted Measurement Study	VAS<br>NRS<br>VRS	121	Ostheoarthritic pain
Jenkins et al., 2009 **[26]**	Correlational Study	VNRS<br>VRS	50	Pruritus
Hicks et al., 2001 **[27]**	Randomized Controlled Trial	FPS		Acute and chronic pain (generic)
Suraseranivongse et al., 2005 **[28]**	Cross-validation Study	FPS<br>VRS<br>CHEOPS	87	Post-operative pain
Sun et al., 2015 **[29]**	Cross-validation Study	FPS-R<br>CAS	62	Pain due to ASA I-III
Gulur et al., 2009 **[30]**	Cross-validation Study	CFPS	129	Acute and chronic pain (generic)
Fadayevatan et al., 2019 **[31]**	Cross-validation Study	FPS	217	Chronic knee pain
Lee et al., 2015 **[32]**	Comparative Study	RAS	60	Chronic spinal pain
Girandeau et al., 2004 **[33]**	Randomized Controlled Trial	VAS	85	Due to sciatica
Ferreira-Valente et al., 2011 **[34]**	Cross-validation Study	VAS<br>NRS<br>VRS<br>FPS-R	127	Experimentally-induced pain
Thong et al., 2018 **[35]**	Cross-validation Study	NRS<br>VAS<br>VRS<br>FPS-R	101	CLBP<br>Knee pain

Miró et al., 2016 **[36]**	Randomized Controlled Trial	NRS<br>VRS<br>FPS	113	Acute and chronic pain (generic)
Malara et al., 2016 **[37]**	Prospective Observational Study	NRS<br>PAINAD<br>CSDD<br>CMAI<br>NPI	233	Acute and chronic pain (generic)
Ersek et al., 2010 **[38]**	Randomized Controlled Trial	CNPI<br>PAINAD	60	Acute and chronic pain (generic)
Paulson-Conger et al., 2011 **[39]**	Descriptive, Comparative, Prospective Study	CPOT<br>PAINAD	100	Acute and chronic pain (generic)
De-Figuerido et al., 2020 **[40]**	Randomized Controlled Trial	VAS<br>VRS<br>PIS	120	Orofacial pain (dental)
Tran et al., 2023 **[41]**	Cross-sectional Study	EDA<br>Cold and electric pulp testing<br>VAS	53	Orofacial pain (dental)
Odai et al., 2015 **[42]**	Cross-sectional Study	VAS<br>FCT	185	Orofacial pain (dental)
Shah et al., 2012 **[43]**	Randomized Controlled Trial	VAS<br>Swelling and trismus assessment Scale	60	Orofacial pain (dental)
Khatri et al., 2012 **[44]**	Comparative Study	VAS<br>FPS	180	Orofacial pain (dental)
Versloot et al., 2004 **[45]**	Randomized Controlled Trial	DDQ	99	Orofacial pain (dental)
Felipak et al., 2020	Cross-sectional Study	DDQ	375	Orofacial pain (dental)

**[46]**				
Daher et al., 2015 **[47]**	Randomized Controlled Trial	DDQ	326	Orofacial pain (dental)
Senirkentli et al., 2021 **[48]**	Cross-sectional Study	DDQ	81	Orofacial pain (dental)
Mendonça et al., 2018 **[49]**	Validation Study	Body maps	80	Muscoloskeletal Pain
Aibel et al., 2023 **[50]**	Validation Study	Pelvic Pain Map GUP)	298	Chronic Pelvic Pain Syndrome (CPPS)
Elson et al., 2011 **[51]**	Validation Study	Photografic Knee Pain Map	70	Knee pain
Adamo et al., 2020 **[52]**	Case-control Clinical Study	OHIP-14<br>GOHAI<br>VAS<br>HAM-A<br>HAM-D	52	Orofacial pain (mucosal)
Sevrain et al., 2015 **[53]**	Retrospective Clinical Study	DN4i<br>HADS<br>QDSA<br>MPQ	35	Orofacial pain (mucosal)
Melzack et al., 1985 **[54]**	Clinical Trial	MPQ	145	Musculoskeletal pain
Kuliś et al., 2011 **[55]**	Validation Study	;MPQ<br>SF-36	30	Musculoskeletal pain
Fontana Carvalho et al., 2020 **[56]**	Pilot Randomized Clinical Trial	MPQ<br>VAS	20	CLBP
Renovato França et al., 2010	Randomized Controlled Trial	MPQ<br>ODQ	30	CLBP

**[57]**				
Dworkin et al., 2015 **[58]**	Secondary Analysis Study	MPQ<br>BPI<br>HADS<br>RMDQ	666	Acute LBP<br>Radicular leg pain
Erdogan et al., 2019 **[59]**	Cross-sectional Study	Cold and electric pulp testing<br>Percussion testing	228	Orofacial pain (dental)
Lewandowski et al., 2009 **[60]**	Randomized Controlled Trial	Pain diary<br>RCADS<br>MDD<br>FPS<br>Children'ss Activity Limitations Interview	93	Orofacial pain (headache)<br>Chronic arthritis pain<br>Due to sicke cell disease
Vertsberger et al., 2022 **[61]**	Observational Study	Pain diary	84	CLBP
Karoly et al., 2014 **[62]**	Observational Study	Interview<br>Pain diary	131	Chronic pain (generic)
Gruszka et al., 2019 **[63]**	Remote app-based Study	Pain diary	205	Acute and chronic pain (generic)
Mitra et al., 2020 **[64]**	Clinical Trial	BPAT	400	Acute and chronic pain (generic)
Delgado et al., 2021 **[65]**	Randomized Controlled Trial	Frankl Behavior Rating Scale	100	Orofacial pain (dental or mucosal)
Gomarverdi et al., 2019 **[66]**	Cross-sectional Study	BPS<br>CPOT	90	Acute and chronic pain (generic)
Ruscheweyh et al., 2012	Validation Study	PSQ	185	Chronic pain (generic)

**[67]**				
Sellers et al., 2013 **[68]**	Observational Study	PSQ<br>VAS	136	LBP
Bell et al., 2018 **[69]**	Comparative Study	PSQ<br>VAS	57	Acute and chronic pain (generic)
Heary et al., 2020 **[70]**	Retrospective Study	PSQ<br>MPQ	331	Due to nerve injury
Müller et al., 2017 **[71]**	Cross-sectional Study	NRS<br>USRP<br>MHI<br>WHOQoL-BREF	834	Due to nerve injury
Kwan et al., 2016 **[72]**	Cross-sectional Study	SF-36	196	Due to spondyloarthritis
Kishi et al., 2005 **[73]**	Cohort Study	SF-36<br>Volatile sulfur compound concentration in mouth air	70	Halitosis
Campos et al., 2021 **[74]**	Two Cross-sectional Studies	OHIP-14	5266	Orofacial pain
Omara et al., 2021 **[75]**	Cross-sectional Study	OHIP-14	516	Orofacial pain<br>Due to ostheoarthritis
Musskopf et al., 2018 **[76]**	Randomized Controlled Trial	OHIP-14	210	Orofacial pain (dental or mucosal)
Yule et al., 2015 **[77]**	Validation Study	RCD/TMD assessment<br>OHIP-49	76	Orofacial pain (temporomandibular)
Serrano et al., 2022 **[78]**	Observational Cross-sectional Study	OHIP-14<br>VAS<br>DMFT<br>Sialometry	61	Due to Sjögren's syndrome

Chana et al., 2021 **[79]**	Cross-sectional Study	PCS<br>PSEQ<br>CPAQ	36	Orofacial pain (mucosal)
Lòpez-Jornet et al., 2008 **[80]**	Observational Study	OHIP-49	60	Orofacial pain
Kyle et al., 2016 **[81]**	Randomized Controlled Trial	HAM-D	86	Acute and chronic pain (generic)
Munhoz Carneiro et al., 2015 **[82]**	Randomized Controlled Trial	HAM-D<br>MADS	91	Acute and chronic pain (generic)
Meltzer-Brody et al., 2009 **[83]**	Clinical Trial	VAS<br>HAM-A<br>HAM-D<br>MPQ	43	Chronic Pelvic Pain
Donham et al., 1984 **[84]**	Cross-validation Study	STAI	219	Acute and chronic pain (generic)
Canfora et al., 2022 **[85]**	Case-control Study	VAS<br>SF-MPQ<br>BPI<br>PD-Q<br>BDI-II<br>STAI<br>PSQI<br>ESS<br>SF-36<br>OHIP-14	40	Orofacial (mucosal)
Zitser et al., 2022 **[86]**	Cross-validation Study	At-home sleep assessment<br>PSQI	32	Acute and chronic pain (generic)

Freedland et al., 2019 **[87]**	Randomized Controlled Trial	BDI<br>PROMIS®	158	Acute and chronic pain
Choi et al., 2014 **[88]**	Prospective Cohort Study	BDI<br>HADS-D<br>PHQ<br>SF-36	546	Due to nerve injury
Chan et al., 2017 **[89]**	Validation Study	HADS-A<br>HADS-D	160	Due to spondyloarthritis
Nipp et al., 2018 **[90]**	Randomized Trial	FACT-G<br>HADS	237	Due to advanced cancer
Mitchell et al., 2010 **[91]**	Validation Study	VAS<br>MPQ<br>FSFI	18	Pelvic pain
Sikora et al., 2018 **[92]**	Observational Case-control Study	STAI<br>BDI	93	Orofacial Pain
Malik et al., 2012 **[93]**	Clinical Trial	VAS<br>HADS<br>GHQ	100	Orofacial Pain
Burns et al., 2012 **[94]**	Randomized Controlled Trial	BPI<br>PCS<br>NRS<br>Roland-Morris Disability Scale<br>Center for Epidemiological Study<br>RMDS<br>Depression Assessment<br>QoL Assessment	83	Chronic pain (generic)

Shi et al., 2022 **[95]**	Cross-sectional Study	TIPI	2223	Acute and chronic pain (generic)
Nunes et al., 2018 **[96]**	Cross-sectional Study	TIPI	333	Acute and chronic pain (generic)
Thørrisen et al., 2021 **[97]**	Cross-sectional Study	TIPI	5009	Acute and chronic pain (generic)
Huyan et al., 2021 **[98]**	Cross-sectional Study	TIPI<br>VAS<br>MPQ<br>Zung's Self-Rating Depression Scale<br>PCS<br>Central Sensitization Inventory	248	Orofacial pain
Darnall et al., 2017 **[99]**	Cross-sectional Validation Study	PCS	519	Chronic pain (generic)
Cano et al., 2005 **[100]**	Preliminary Validation Study	PCS<br>NRS	264	Acute and chronic pain (generic)
Roguli et al., 2014 **[101]**	Cross-sectional Study	OHIP-14<br>PCS	30	Orofacial
Walker et al., 2020 **[102]**	Retrospective Cohort Study	ESS	85	Acute and chronic pain (generic)
Sap-Anan et al., 2021 **[103]**	Validation Study	ESS<br>BDI<br>In-laboratory polysomnography<br>ISI	95	Acute and chronic pain (generic)
Frohnhofen et al., 2009	Randomized Controlled Trial	ESS	458	Acute and chronic pain (generic)

**[104]**				
Damiani et al., 2013 **[105]**	Randomized Controlled Trial	ESS	225	Acute and chronic pain (generic)
Adamo et al., 2018 **[106]**	Case-Control Multicenter Study	PSQI<br>ESS<br>HAM-D<br>HAM-A<br>NRS<br>T-PRI	200	Orofacial Pain
Kirmizigil et al., 2020 **[107]**	Randomized Controlled Trial	VAS<br>MSQ<br>PSQI	28	Pelvic Pain
Lee et al., 2020 **[108]**	Cross-sectional Study	NRS<br>PSQI<br>HADS	25	Orofacial Pain
Lee et al., 2022 **[109]**	Cross-sectional Study	BPI<br>PSQI<br>SCL-90	65	Orofacial Pain
Lopez-Jornet et al., 2014 **[110]**	Observational Study	VAS<br>HADS<br>OHIP-14<br>PSQI<br>ESS	70	Orofacial Pain

Abbreviations (in alphabetical order): BAI, Beck's anxiety scale; BPI, brief pain inventory; CAS, color analogue scale; CMAI, Cohen-Mansfield agitation inventory; CNPI, checklist of nonverbal pain behaviors; CPAQ, chronic pain acceptance questionnaire; CPOT, critical-care pain observation tool; CSDD, Cornell scale for depression in dementia; CVAS, computerized visual analogue scale; DDQ, dental discomfort questionnaire; DMFT, decayed missing filled teeth; DN4i, douleur neuropathique 4-questionnaire; EDA, electrodermal activity; EIHS, Edinburgh inventory handedness scale; ESS, Epsworth sleepiness scale; FCT, full-cup test; FBRS, Frank's behavior rating scale; FPS, faces pain scale; FPS-R, faces pain scale revised; FACT-G, functional assessment of cancer therapy-general; FMRIB, functional magnetic resonance imaging of the brain; GHQ, general health questionnaire; GOHAI, general oral-health assessment index; GUPI, genitourinary pain index; HAM-A, Hamilton's anxiety rating scale; HAM-D, Hamilton's depression rating scale; HADS, hospital anxiety and depression scale; ISI, insomnia severity index; MADS, Montgomery-Asber

depression scale; MDD, major depressive disorder subscale; MPQ, McGill pain questionnaire; MSQ, menstrual symptoms questionnaire; MVAS, mechanical visual analogue scale; NPI, neuropsychiatric inventory; NPS, Washington neuropathic pain scale; NRS, numerical rating scale; ODQ, Oswestry disability questionnaire; OHIP-14, oral-health impact profile-14; PAINAD, pain assessment in advanced dementia; PD-Q, Pain DETECT questionnaire; PCS, pain catastrophizing scale; PHQ, patient health questionnaire depression module; PIS, periapical index score; PQDSA, questionnaire de la douleur de Saint-Antoine; PROMIS®, patient-reported outcomes measurement information system®; QDSA, questionnaire de la douleur de Saint-Antoine; RCADS, revised child anxiety and depression scale; RCD/TMD, research diagnostic criteria/temporomandibular disorders; RMDS, Roland-Morris disability scale center for epidemiological study; SF-36, short-form 36; SF-MPQ, short-form McGill pain questionnaire; SCL-90, symptoms checklist-90; STAI, state-trait anxiety index; T-PRI, total pain rating index; TIPI, ten-item personality inventory; USRP, Utrecht's scale of evaluation of participation; VAS, visual analogue scale; VNRS, verbal numeric rating scale; VRS, verbal rating scale; ZSDP, Zung's self-rating depression scale

Table S4. Criteria for judging risk of bias in the "Risk of bias" assessment tool.

**Random Sequence Generation**	
Criteria for a judgement of 'Low risk' of bias.	The investigators describe a random component in the sequence generation process.
Criteria for the judgement of 'High risk' of bias.	The investigators describe a non-random component in the sequence generation process. Usually, the description would involve some systematic, non-random approach.<br>Other non-random approaches happen much less frequently than the systematic approaches mentioned above and tend to be obvious. They usually involve judgement or some method of non-random categorization of participants.
**Allocation Concealment**	
Criteria for a judgement of 'Low risk' of bias.	Participants and investigators enrolling participants could not foresee assignment because one of the following, or an equivalent method, was used to conceal allocation.
Criteria for the judgement of 'High risk' of bias.	Participants or investigators enrolling participants could possibly foresee assignments and thus introduce selection bias.
**Blinding**	
Criteria for a judgement of 'Low risk' of bias.	Any one of the following:<br>- No blinding or incomplete blinding, but the review authors judge that the outcome is not likely to be influenced by lack of blinding;<br>- Blinding of participants and key study personnel ensured, and unlikely that the blinding could have been broken;<br>- No blinding of outcome assessment, but the review authors judge that the outcome measurement is not likely to be influenced by lack of blinding;<br>- Blinding of outcome assessment ensured, and unlikely that the blinding could have been broken.
Criteria for the judgement of 'High risk' of bias.	Any one of the following:<br>- No blinding or incomplete blinding, and the outcome is likely to be influenced by lack of blinding;

	- Blinding of key study participants and personnel attempted, but likely that the blinding could have been broken, and the outcome is likely to be influenced by lack of blinding;<br>- No blinding of outcome assessment, and the outcome measurement is likely to be influenced by lack of blinding;<br>- Blinding of outcome assessment, but likely that the blinding could have been broken, and the outcome measurement is likely to be influenced by lack of blinding.
**Incomplete Outcome Data**	
Criteria for a judgement of 'Low risk' of bias.	Any one of the following:<br>- No missing outcome data;<br>- Reasons for missing outcome data unlikely to be related to true outcome (for survival data, censoring unlikely to be introducing bias);<br>- Missing outcome data balanced in numbers across intervention groups, with similar reasons for missing data across groups;<br>- For dichotomous outcome data, the proportion of missing outcomes compared with observed event risk not enough to have a clinically relevant impact on the intervention effect estimate;<br>- For continuous outcome data, plausible effect size (difference in means or standardized difference in means) among missing outcomes not enough to have a clinically relevant impact on observed effect size;<br>- Missing data have been imputed using appropriate methods.
Criteria for the judgement of 'High risk' of bias.	Any one of the following:<br>- Reason for missing outcome data likely to be related to true outcome, with either imbalance in numbers or reasons for missing data across intervention groups;<br>- For dichotomous outcome data, the proportion of missing outcomes compared with observed event risk enough to induce clinically relevant bias in intervention effect estimate;<br>- For continuous outcome data, plausible effect size (difference in means or standardized difference in means) among missing outcomes enough to induce clinically relevant bias in observed effect size;

	- 'As-treated' analysis done with substantial departure of the intervention received from that assigned at randomization;<br>- Potentially inappropriate application of simple imputation.
**Selective Reporting**	
Criteria for a judgement of 'Low risk' of bias.	Any one of the following:<br>- The study protocol is available and all of the study's pre-specified (primary and secondary) outcomes that are of interest in the review have been reported in the pre-specified way;<br>- The study protocol is not available but it is clear that the published reports include all expected outcomes, including those that were pre-specified (convincing text of this nature may be uncommon).
Criteria for the judgement of 'High risk' of bias.	Any one of the following:<br>- Not all of the study's pre-specified primary outcomes have been reported;<br>- One or more primary outcomes is reported using measurements, analysis methods or subsets of the data (e.g., subscales) that were not pre-specified;<br>- One or more reported primary outcomes were not pre-specified (unless clear justification for their reporting is provided, such as an unexpected adverse effect);<br>- One or more outcomes of interest in the review are reported incompletely so that they cannot be entered in a meta-analysis;<br>- The study report fails to include results for a key outcome that would be expected to have been reported for such a study.

References

1. Menendez ME, Ring D. Factors Associated with Greater Pain Intensity. Hand Clin. 2016 Feb;32(1):27-31. doi: 10.1016/j.hcl.2015.08.004. PMID: 26611386.
2. Hawker GA, Mian S, Kendzerska T, French M. Measures of adult pain: Visual Analog Scale for Pain (VAS Pain), Numeric Rating Scale for Pain (NRS Pain), McGill Pain Questionnaire (MPQ), Short-Form McGill Pain Questionnaire (SF-MPQ), Chronic Pain Grade Scale (CPGS), Short Form-36 Bodily Pain Scale (SF-36 BPS), and Measure of Intermittent and Constant Osteoarthritis Pain (ICOAP). Arthritis Care Res (Hoboken). 2011 Nov;63 Suppl 11:S240-52. doi: 10.1002/acr.20543. PMID: 22588748.
3. Hajihasani A, Rouhani M, Salavati M, Hedayati R, Kahlaee AH. The Influence of Cognitive Behavioral Therapy on Pain, Quality of Life, and Depression in Patients Receiving Physical Therapy for Chronic Low Back Pain: A Systematic Review. PM R. 2019 Feb;11(2):167-176. doi: 10.1016/j.pmrj.2018.09.029. Epub 2019 Feb 11. PMID: 30266349.
4. Khalid W. Questionnaire designing and validation. J Pak Med Assoc. 2012 May;62(5):514-6. PMID: 22755326.
5. Xu X, Huang Y. Objective Pain Assessment: a Key for the Management of Chronic Pain. F1000Res. 2020 Jan 23;9:F1000 Faculty Rev-35. doi: 10.12688/f1000research.20441.1. PMID: 32047606; PMCID: PMC6979466. Kazi AM,
6. Raja SN, Carr DB, Cohen M, Finnerup NB, Flor H, Gibson S, Keefe FJ, Mogil JS, Ringkamp M, Sluka KA, Song XJ, Stevens B, Sullivan MD, Tutelman PR, Ushida T, Vader K. The revised International Association for the Study of Pain definition of pain: concepts, challenges, and compromises. Pain. 2020 Sep 1;161(9):1976-1982. doi: 10.1097/j.pain.0000000000001939. PMID: 32694387; PMCID: PMC7680716.
7. Mercer Lindsay N, Chen C, Gilam G, Mackey S, Scherrer G. Brain circuits for pain and its treatment. Sci Transl Med. 2021 Nov 10;13(619):eabj7360. doi: 10.1126/scitranslmed.abj7360. Epub 2021 Nov 10. PMID: 34757810; PMCID: PMC8675872.
8. Gorczyca R, Filip R, Walczak E. Psychological aspects of pain. Ann Agric Environ Med. 2013;Spec no. 1:23-7. PMID: 25000837.
9. International Classification of Orofacial Pain, 1st edition (ICOP). Cephalalgia. 2020;40(2):129-221. doi:10.1177/0333102419893823
10. Lin Y, De Araujo I, Stanley G, Small D, Geha P. Chronic pain precedes disrupted eating behavior in low-back pain patients. PLoS One. 2022 Feb 10;17(2):e0263527. doi: 10.1371/journal.pone.0263527. PMID: 35143525; PMCID: PMC8830732.
11. Sheng J, Liu S, Wang Y, Cui R, Zhang X. The Link between Depression and Chronic Pain: Neural Mechanisms in the Brain. Neural Plast. 2017;2017:9724371. doi: 10.1155/2017/9724371. Epub 2017 Jun 19. PMID: 28706741; PMCID: PMC5494581.
12. Michaelides A, Zis P. Depression, anxiety and acute pain: links and management challenges. Postgrad Med. 2019 Sep;131(7):438-444. doi: 10.1080/00325481.2019.1663705. Epub 2019 Sep 12. PMID: 31482756.
13. Rogers AH, Farris SG. A meta-analysis of the associations of elements of the fear-avoidance model of chronic pain with negative affect, depression, anxiety, pain-related disability and pain intensity. Eur J Pain. 2022 Sep;26(8):1611-1635. doi: 10.1002/ejp.1994. Epub 2022 Jul 7. PMID: 35727200; PMCID: PMC9541898.
14. Finan PH, Goodin BR, Smith MT. The association of sleep and pain: an update and a path forward. J Pain. 2013 Dec;14(12):1539-52. doi: 10.1016/j.jpain.2013.08.007. PMID: 24290442; PMCID: PMC4046588.
15. Whibley D, AlKandari N, Kristensen K, Barnish M, Rzewuska M, Druce KL, Tang NKY. Sleep and Pain: A Systematic Review of Studies of Mediation. Clin J Pain. 2019 Jun;35(6):544-558. doi: 10.1097/AJP.0000000000000697. PMID: 30829737; PMCID: PMC6504189.
16. Treister R, Honigman L, Lawal OD, Lanier RK, Katz NP. A deeper look at pain variability and its relationship with the placebo response: results from a randomized, double-blind, placebo-controlled clinical trial of naproxen in osteoarthritis of the knee. Pain. 2019 Jul;160(7):1522-1528. doi: 10.1097/j.pain.0000000000001538. PMID: 30817436.

17. Shafshak TS, Elnemr R. The Visual Analogue Scale Versus Numerical Rating Scale in Measuring Pain Severity and Predicting Disability in Low Back Pain. J Clin Rheumatol. 2021 Oct 1;27(7):282-285. doi: 10.1097/RHU.0000000000001320. PMID: 31985722.
18. Dawn B. Kendrick, Tania D. Strout, The minimum clinically significant difference in patient-assigned numeric scores for pain, The American Journal of Emergency Medicine, Volume 23, Issue 7, 2005, Pages 828-832, ISSN 0735-6757, https://doi.org/10.1016/j.ajem.2005.07.009. (https://www.sciencedirect.com/science/article/pii/S0735675705002433)
19. Taddio A, O'Brien L, Ipp M, Stephens D, Goldbach M, Koren G. Reliability and validity of observer ratings of pain using the visual analog scale (VAS) in infants undergoing immunization injections. Pain. 2009 Dec 15;147(1-3):141-6. doi: 10.1016/j.pain.2009.08.027. Epub 2009 Sep 19. PMID: 19767147.
20. Todd KH, Funk KG, Funk JP, Bonacci R. Clinical significance of reported changes in pain severity. Ann Emerg Med. 1996 Apr;27(4):485-9. doi: 10.1016/s0196-0644(96)70238-x. PMID: 8604867.
21. Closs SJ, Barr B, Briggs M, Cash K, Seers K. A comparison of five pain assessment scales for nursing home residents with varying degrees of cognitive impairment. J Pain Symptom Manage. 2004 Mar;27(3):196-205. doi: 10.1016/j.jpainsymman.2003.12.010. PMID: 15010098.
22. Lewinson RT, Wiley JP, Worobets JT, Stefanyshyn DJ. Development and validation of a computerized visual analog scale for the measurement of pain in patients with patellofemoral pain syndrome. Clin J Sport Med. 2013 Sep;23(5):392-6. doi: 10.1097/JSM.0b013e31828b0848. PMID: 23624572.
23. Ruskin D, Lalloo C, Amaria K, Stinson JN, Kewley E, Campbell F, Brown SC, Jeavons M, McGrath PA. Assessing pain intensity in children with chronic pain: convergent and discriminant validity of the 0 to 10 numerical rating scale in clinical practice. Pain Res Manag. 2014 May-Jun;19(3):141-8. doi: 10.1155/2014/856513. Epub 2014 Apr 7. PMID: 24712019; PMCID: PMC4158959.
24. Wikström L, Nilsson M, Broström A, Eriksson K. Patients' self-reported nausea: Validation of the Numerical Rating Scale and of a daily summary of repeated Numerical Rating Scale scores. J Clin Nurs. 2019 Mar;28(5-6):959-968. doi: 10.1111/jocn.14705. Epub 2018 Nov 8. PMID: 30357970.
25. Alghadir AH, Anwer S, Iqbal A, Iqbal ZA. Test-retest reliability, validity, and minimum detectable change of visual analog, numerical rating, and verbal rating scales for measurement of osteoarthritic knee pain. J Pain Res. 2018 Apr 26;11:851-856. doi: 10.2147/JPR.S158847. PMID: 29731662; PMCID: PMC5927184.
26. Jenkins HH, Spencer ED, Weissgerber AJ, Osborne LA, Pellegrini JE. Correlating an 11-point verbal numeric rating scale to a 4-point verbal rating scale in the measurement of pruritus. J Perianesth Nurs. 2009 Jun;24(3):152-5. doi: 10.1016/j.jopan.2009.01.010. PMID: 19500747.
27. Hicks CL, von Baeyer CL, Spafford PA, van Korlaar I, Goodenough B. The Faces Pain Scale-Revised: toward a common metric in pediatric pain measurement. Pain. 2001 Aug;93(2):173-183. doi: 10.1016/S0304-3959(01)00314-1. PMID: 11427329.
28. Suraseranivongse S, Montapaneewat T, Manon J, Chainchop P, Petcharatana S, Kraiprasit K. Cross-validation of a self-report scale for postoperative pain in school-aged children. J Med Assoc Thai. 2005 Mar;88(3):412-8. PMID: 15962653.
29. Sun T, West N, Ansermino JM, Montgomery CJ, Myers D, Dunsmuir D, Lauder GR, von Baeyer CL. A smartphone version of the Faces Pain Scale-Revised and the Color Analog Scale for postoperative pain assessment in children. Paediatr Anaesth. 2015 Dec;25(12):1264-73. doi: 10.1111/pan.12790. PMID: 26507916.
30. Cravero JP, Fanciullo GJ, McHugo GJ, Baird JC. The validity of the Computer Face Scale for measuring pediatric pain and mood. Paediatr Anaesth. 2013 Feb;23(2):156-61. doi: 10.1111/pan.12036. Epub 2012 Oct 13. PMID: 23061715.

31. Fadayevatan R, Alizadeh-Khoei M, Hessami-Azar ST, Sharifi F, Haghi M, Kaboudi B. Validity and Reliability of 11-face Faces Pain Scale in the Iranian Elderly Community with Chronic Pain. Indian J Palliat Care. 2019 Jan-Mar;25(1):46-51. doi: 10.4103/IJPC.IJPC_126_18. PMID: 30820100; PMCID: PMC6388585.
32. Lee JJ, Lee MK, Kim JE, Kim HZ, Park SH, Tae JH, Choi SS. Pain relief scale is more highly correlated with numerical rating scale than with visual analogue scale in chronic pain patients. Pain Physician. 2015 Mar-Apr;18(2):E195-200. PMID: 25794219
33. Giraudeau B, Rozenberg S, Valat JP. Assessment of the clinically relevant change in pain for patients with sciatica. Ann Rheum Dis. 2004 Sep;63(9):1180-1. doi: 10.1136/ard.2003.015792. PMID: 15308536; PMCID: PMC1755131.
34. Ferreira-Valente MA, Pais-Ribeiro JL, Jensen MP. Validity of four pain intensity rating scales. Pain. 2011 Oct;152(10):2399-2404. doi: 10.1016/j.pain.2011.07.005. PMID: 21856077.
35. Thong ISK, Jensen MP, Miró J, Tan G. The validity of pain intensity measures: what do the NRS, VAS, VRS, and FPS-R measure? Scand J Pain. 2018 Jan 26;18(1):99-107. doi: 10.1515/sjpain-2018-0012. PMID: 29794282.
36. Miró J, Castarlenas E, de la Vega R, Solé E, Tomé-Pires C, Jensen MP, Engel JM, Racine M. Validity of three rating scales for measuring pain intensity in youths with physical disabilities. Eur J Pain. 2016 Jan;20(1):130-7. doi: 10.1002/ejp.704. Epub 2015 Mar 31. PMID: 25833415; PMCID: PMC4591090.
37. Malara A, De Biase GA, Bettarini F, Ceravolo F, Di Cello S, Garo M, Praino F, Settembrini V, Sgrò G, Spadea F, Rispoli V. Pain Assessment in Elderly with Behavioral and Psychological Symptoms of Dementia. J Alzheimers Dis. 2016;50(4):1217-25. doi: 10.3233/JAD-150808. PMID: 26757042; PMCID: PMC4927851.
38. Ersek M, Herr K, Neradilek MB, Buck HG, Black B. Comparing the psychometric properties of the Checklist of Nonverbal Pain Behaviors (CNPI) and the Pain Assessment in Advanced Dementia (PAIN-AD) instruments. Pain Med. 2010 Mar;11(3):395-404. doi: 10.1111/j.1526-4637.2009.00787.x. Epub 2010 Jan 15. PMID: 20088854; PMCID: PMC2866060.
39. Paulson-Conger M, Leske J, Maidl C, Hanson A, Dziadulewicz L. Comparison of two pain assessment tools in nonverbal critical care patients. Pain Manag Nurs. 2011 Dec;12(4):218-24. doi: 10.1016/j.pmn.2010.05.008. Epub 2010 Sep 15. PMID: 22117753.
40. de-Figueiredo FED, Lima LF, Lima GS, Oliveira LS, Ribeiro MA, Brito-Junior M, Correa MB, Sousa-Neto MD, Faria E Silva AL. Apical periodontitis healing and postoperative pain following endodontic treatment with a reciprocating single-file, single-cone approach: A randomized controlled pragmatic clinical trial. PLoS One. 2020 Feb 3;15(2):e0227347. doi: 10.1371/journal.pone.0227347. Erratum in: PLoS One. 2020 Mar 11;15(3):e0230511. PMID: 32012166; PMCID: PMC6996828.
41. Tran HT, Kong Y, Talati A, Posada-Quintero H, Chon KH, Chen IP. The use of electrodermal activity in pulpal diagnosis and dental pain assessment. Int Endod J. 2023 Mar;56(3):356-368. doi: 10.1111/iej.13868. Epub 2022 Nov 17. PMID: 36367715; PMCID: PMC10044487.
42. Odai ED, Ehizele AO, Enabulele JE. Assessment of pain among a group of Nigerian dental patients. BMC Res Notes. 2015 Jun 19;8:251. doi: 10.1186/s13104-015-1226-5. PMID: 26087661; PMCID: PMC4474451.
43. Shah R, Mahajan A, Shah N, Dadhania AP. Preemptive analgesia in third molar impaction surgery. Natl J Maxillofac Surg. 2012 Jul;3(2):144-7. doi: 10.4103/0975-5950.111368. PMID: 23833488; PMCID: PMC3700147.
44. Khatri A, Kalra N. A comparison of two pain scales in the assessment of dental pain in East delhi children. ISRN Dent. 2012;2012:247351. doi: 10.5402/2012/247351. Epub 2012 Feb 14. PMID: 22461986; PMCID: PMC3302111.
45. Versloot J, Veerkamp JS, Hoogstraten J. Dental Discomfort Questionnaire: predicting toothache in preverbal children. Eur J Paediatr Dent. 2004 Sep;5(3):170-3. PMID: 15471526.

46. Felipak PK, Menoncin BLV, Reyes MRT, Costa LR, de Souza JF, Menezes JVNB. Determinants of parental report of dental pain and discomfort in preschool children-The Dental Discomfort Questionnaire. Int J Paediatr Dent. 2020 Jul;30(4):436-444. doi: 10.1111/ipd.12614. Epub 2020 Jan 24. PMID: 31900956.
47. Daher A, Abreu MH, Costa LR. Recognizing preschool children with primary teeth needing dental treatment because of caries-related toothache. Community Dent Oral Epidemiol. 2015 Aug;43(4):298-307. doi: 10.1111/cdoe.12154. Epub 2015 Feb 6. PMID: 25656813.
48. Senirkentli GB, Tirali RE, Bani M. Assessment of dental pain in children with intellectual disability using the dental discomfort questionnaire. J Intellect Disabil. 2021 Jan 27:1744629520981318. doi: 10.1177/1744629520981318. Epub ahead of print. PMID: 33499707.
49. Mendonça CR, Noll M, Silveira EA. Adaptation and validation of body maps for musculoskeletal pain location in patients with severe obesity. Korean J Pain. 2018 Oct;31(4):268-276. doi: 10.3344/kjp.2018.31.4.268. Epub 2018 Oct 1. PMID: 30310552; PMCID: PMC6177535.
50. Aibel K, Moldwin R. Validation of the 'Pelvic Pain Map': a new self-assessment tool for chronic pelvic pain localisation. BJU Int. 2023 Jun;131(6):763-769. doi: 10.1111/bju.15979. Epub 2023 Mar 23. PMID: 36722397.
51. Elson DW, Jones S, Caplan N, Stewart S, St Clair Gibson A, Kader DF. The photographic knee pain map: locating knee pain with an instrument developed for diagnostic, communication and research purposes. Knee. 2011 Dec;18(6):417-23. doi: 10.1016/j.knee.2010.08.012. Epub 2010 Sep 17. PMID: 20850976.
52. Adamo D, Pecoraro G, Fortuna G, Amato M, Marenzi G, Aria M, Mignogna MD. Assessment of oral health-related quality of life, measured by OHIP-14 and GOHAI, and psychological profiling in burning mouth syndrome: A case-control clinical study. J Oral Rehabil. 2020 Jan;47(1):42-52. doi: 10.1111/joor.12864. Epub 2019 Aug 10. PMID: 31332814.
53. Sevrain M, Brenaut E, Le Toux G, Misery L. Primary Burning Mouth Syndrome: A Questionnaire Study of Neuropathic and Psychological Components. Am J Clin Dermatol. 2016 Apr;17(2):171-8. doi: 10.1007/s40257-015-0170-4. PMID: 26691521.
54. Melzack R, Katz J, Jeans ME. The role of compensation in chronic pain: analysis using a new method of scoring the McGill Pain Questionnaire. Pain. 1985 Oct;23(2):101-112. doi: 10.1016/0304-3959(85)90052-1. PMID: 2933623.
55. Kachooei AR, Ebrahimzadeh MH, Erfani-Sayyar R, Salehi M, Salimi E, Razi S. Short Form-McGill Pain Questionnaire-2 (SF-MPQ-2): A Cross-Cultural Adaptation and Validation Study of the Persian Version in Patients with Knee Osteoarthritis. Arch Bone Jt Surg. 2015 Jan;3(1):45-50. Epub 2015 Jan 15. PMID: 25692169; PMCID: PMC4322125..
56. Fontana Carvalho AP, Dufresne SS, Rogerio de Oliveira M, Couto Furlanetto K, Dubois M, Dallaire M, Ngomo S, da Silva RA. Effects of lumbar stabilization and muscular stretching on pain, disabilities, postural control and muscle activation in pregnant woman with low back pain. Eur J Phys Rehabil Med. 2020 Jun;56(3):297-306. doi: 10.23736/S1973-9087.20.06086-4. Epub 2020 Feb 18. PMID: 32072792.
57. França FR, Burke TN, Hanada ES, Marques AP. Segmental stabilization and muscular strengthening in chronic low back pain: a comparative study. Clinics (Sao Paulo). 2010;65(10):1013-7. doi: 10.1590/s1807-59322010001000015. PMID: 21120303; PMCID: PMC2972594.
58. Dworkin RH, Turk DC, Trudeau JJ, Benson C, Biondi DM, Katz NP, Kim M. Validation of the Short-form McGill Pain Questionnaire-2 (SF-MPQ-2) in acute low back pain. J Pain. 2015 Apr;16(4):357-66. doi: 10.1016/j.jpain.2015.01.012. Epub 2015 Jan 29. PMID: 25640290.
59. Erdogan O, Malek M, Janal MN, Gibbs JL. Sensory testing associates with pain quality descriptors during acute dental pain. Eur J Pain. 2019 Oct;23(9):1701-1711. doi: 10.1002/ejp.1447. Epub 2019 Jul 22. PMID: 31241807.
60. Lewandowski AS, Palermo TM, Kirchner HL, Drotar D. Comparing diary and retrospective reports of pain and activity restriction in children and adolescents with chronic pain conditions. Clin J Pain. 2009 May;25(4):299-306. doi: 10.1097/AJP.0b013e3181965578. PMID: 19590478; PMCID: PMC2709738.

61. Vertsberger D, Talmon A, Ziadni M, Kong JT, Darnall BD, Manber R, Mackey S, Gross JJ. Intensity of Chronic Low Back Pain and Activity Interference: A Daily Diary Study of the Moderating Role of Cognitive Pain Coping Strategies. Pain Med. 2023 Apr 3;24(4):442-450. doi: 10.1093/pm/pnac151. PMID: 36214626; PMCID: PMC10069845.
62. Karoly P, Okun MA, Enders C, Tennen H. Effects of pain intensity on goal schemas and goal pursuit: a daily diary study. Health Psychol. 2014 Sep;33(9):968-76. doi: 10.1037/hea0000093. PMID: 25180547; PMCID: PMC4155604.
63. Gruszka P, Stammen C, Bissantz N, Jensen MP. Pain vs. comfort diary: A fully remote app-based experiment. Eur J Pain. 2019 Oct;23(9):1674-1687. doi: 10.1002/ejp.1446. Epub 2019 Jun 24. PMID: 31233662.
64. Mitra S, Jain K, Singh J, Saxena P, Nyima T, Selvam SR, Walia MC. Clinical Utility of the Behavioral Pain Assessment Tool in Patients Admitted in the Intensive Care Unit. Indian J Crit Care Med. 2020 Aug;24(8):695-700. doi: 10.5005/jp-journals-10071-23521. PMID: 33024377; PMCID: PMC7519617.
65. Delgado A, Ok SM, Ho D, Lynd T, Cheon K. Evaluation of children's pain expression and behavior using audio visual distraction. Clin Exp Dent Res. 2021 Oct;7(5):795-802. doi: 10.1002/cre2.407. Epub 2021 Feb 23. PMID: 33622030; PMCID: PMC8543459.
66. Gomarverdi S, Sedighie L, Seifrabiei MA, Nikooseresht M. Comparison of Two Pain Scales: Behavioral Pain Scale and Critical-care Pain Observation Tool During Invasive and Noninvasive Procedures in Intensive Care Unit-admitted Patients. Iran J Nurs Midwifery Res. 2019 Mar-Apr;24(2):151-155. doi: 10.4103/ijnmr.IJNMR_47_18. PMID: 30820228; PMCID: PMC6390431.
67. Ruscheweyh R, Verneuer B, Dany K, Marziniak M, Wolowski A, Çolak-Ekici R, Schulte TL, Bullmann V, Grewe S, Gralow I, Evers S, Knecht S. Validation of the pain sensitivity questionnaire in chronic pain patients. Pain. 2012 Jun;153(6):1210-1218. doi: 10.1016/j.pain.2012.02.025. Epub 2012 Apr 25. PMID: 22541722.
68. Sellers AB, Ruscheweyh R, Kelley BJ, Ness TJ, Vetter TR. Validation of the English language pain sensitivity questionnaire. Reg Anesth Pain Med. 2013 Nov-Dec;38(6):508-14. doi: 10.1097/AAP.0000000000000007. PMID: 24141873.
69. Bell BA, Ruscheweyh R, Kelley BJ, Ness TJ, Vetter TR, Sellers AB. Ethnic Differences Identified by Pain Sensitivity Questionnaire Correlate With Clinical Pain Responses. Reg Anesth Pain Med. 2018 Feb;43(2):200-204. doi: 10.1097/AAP.0000000000000689. PMID: 29278602.
70. Heary KO, Wong AWK, Lau SCL, Dengler J, Thompson MR, Crock LW, Novak CB, Philip BA, Mackinnon SE. Quality of Life and Psychosocial Factors as Predictors of Pain Relief Following Nerve Surgery. Hand (N Y). 2022 Mar;17(2):193-199. doi: 10.1177/1558944720911213. Epub 2020 Mar 19. PMID: 32188297; PMCID: PMC8984711.
71. Müller R, Landmann G, Béchir M, Hinrichs T, Arnet U, Jordan X, Brinkhof MWG. Chronic pain, depression and quality of life in individuals with spinal cord injury: Mediating role of participation. J Rehabil Med. 2017 Jun 28;49(6):489-496. doi: 10.2340/16501977-2241. PMID: 28597908.
72. Kwan YH, Fong WW, Lui NL, Yong ST, Cheung YB, Malhotra R, Østbye T, Thumboo J. Validity and reliability of the Short Form 36 Health Surveys (SF-36) among patients with spondyloarthritis in Singapore. Rheumatol Int. 2016 Dec;36(12):1759-1765. doi: 10.1007/s00296-016-3567-3. Epub 2016 Sep 23. PMID: 27664139.
73. Kishi M, Abe A, Yonemitsu M. Relationship between the SF-36 questionnaire and patient's satisfaction following halitosis therapy. Oral Dis. 2005;11 Suppl 1:89-91. doi: 10.1111/j.1601-0825.2005.01102.x. PMID: 15752110.
74. Campos LA, Peltomäki T, Marôco J, Campos JADB. Use of Oral Health Impact Profile-14 (OHIP-14) in Different Contexts. What Is Being Measured? Int J Environ Res Public Health. 2021 Dec 20;18(24):13412. doi: 10.3390/ijerph182413412. PMID: 34949018; PMCID: PMC8703465.

75. Omara M, Salzberger T, Boecker M, Bekes K, Steiner G, Nell-Duxneuner V, Ritschl V, Mosor E, Kloppenburg M, Sautner J, Steinecker-Frohnwieser B, Stamm T. Improving the measurement of oral health-related quality of life: Rasch model of the oral health impact profile-14. J Dent. 2021 Nov;114:103819. doi: 10.1016/j.jdent.2021.103819. Epub 2021 Sep 21. PMID: 34560224.
76. Musskopf ML, Milanesi FC, Rocha JMD, Fiorini T, Moreira CHC, Susin C, Rösing CK, Weidlich P, Oppermann RV. Oral health related quality of life among pregnant women: a randomized controlled trial. Braz Oral Res. 2018;32:e002. doi: 10.1590/1807-3107bor-2018.vol32.0002. Epub 2018 Jan 22. PMID: 29364329.
77. Yule PL, Durham J, Playford H, Moufti MA, Steele J, Steen N, Wassell RW, Ohrbach R. OHIP-TMDs: a patient-reported outcome measure for temporomandibular disorders. Community Dent Oral Epidemiol. 2015 Oct;43(5):461-70. doi: 10.1111/cdoe.12171. Epub 2015 Jun 4. PMID: 26040190.
78. Serrano J, López-Pintor RM, Fernández-Castro M, Ramírez L, Sanz M, López J, Blázquez MÁ, González JJ, Hernández G; EPOX-SSp Group. Usefulness of implementing the OHIP-14 questionnaire to assess the impact of xerostomia and hyposalivation on quality of life in patients with primary Sjögren's syndrome. J Oral Pathol Med. 2022 Oct;51(9):810-817. doi: 10.1111/jop.13348. Epub 2022 Sep 30. PMID: 35998227; PMCID: PMC9828657.
79. Chana P, Smith JG, Karamat A, Simpson A, Renton T. Catastrophising, pain self-efficacy and acceptance in patients with Burning Mouth Syndrome. J Oral Rehabil. 2021 Apr;48(4):458-468. doi: 10.1111/joor.13136. Epub 2021 Jan 5. PMID: 33368621.
80. López-Jornet P, Camacho-Alonso F, Lucero-Berdugo M. Quality of life in patients with burning mouth syndrome. J Oral Pathol Med. 2008 Aug;37(7):389-94. doi: 10.1111/j.1600-0714.2008.00672.x. Epub 2008 Jul 9. PMID: 18665968.
81. Kyle PR, Lemming OM, Timmerby N, Søndergaard S, Andreasson K, Bech P. The Validity of the Different Versions of the Hamilton Depression Scale in Separating Remission Rates of Placebo and Antidepressants in Clinical Trials of Major Depression. J Clin Psychopharmacol. 2016 Oct;36(5):453-6. doi: 10.1097/JCP.0000000000000557. PMID: 27525966.
82. Carneiro AM, Fernandes F, Moreno RA. Hamilton depression rating scale and montgomery-asberg depression rating scale in depressed and bipolar I patients: psychometric properties in a Brazilian sample. Health Qual Life Outcomes. 2015 Apr 2;13:42. doi: 10.1186/s12955-015-0235-3. PMID: 25889742; PMCID: PMC4391145.
83. Meltzer-Brody SE, Zolnoun D, Steege JF, Rinaldi KL, Leserman J. Open-label trial of lamotrigine focusing on efficacy in vulvodynia. J Reprod Med. 2009 Mar;54(3):171-8. PMID: 19370903; PMCID: PMC4676413.
84. Donham GW, Ludenia K. Cross-validation of the State-Trait Anxiety Inventory with an alcoholic population. J Clin Psychol. 1984 Mar;40(2):629-31. doi: 10.1002/1097-4679(198403)40:2<629::aid-jclp2270400244>3.0.co;2-a. PMID: 6725591.
85. Canfora F, Calabria E, Pecoraro G, D Aniello L, Aria M, Marenzi G, Sammartino P, Mignogna MD, Adamo D. The use of self-report questionnaires in an analysis of the multidimensional aspects of pain and a correlation with the psychological profile and quality of life in patients with burning mouth syndrome: A case-control study. J Oral Rehabil. 2022 Sep;49(9):890-914. doi: 10.1111/joor.13343. Epub 2022 Jun 21. PMID: 35611463; PMCID: PMC9544557.
86. Zitser J, Allen IE, Falgàs N, Le MM, Neylan TC, Kramer JH, Walsh CM. Pittsburgh Sleep Quality Index (PSQI) responses are modulated by total sleep time and wake after sleep onset in healthy older adults. PLoS One. 2022 Jun 24;17(6):e0270095. doi: 10.1371/journal.pone.0270095. PMID: 35749529; PMCID: PMC9232154.
87. Freedland KE, Steinmeyer BC, Carney RM, Rubin EH, Rich MW. Use of the PROMIS® Depression scale and the Beck Depression Inventory in patients with heart failure. Health Psychol. 2019 May;38(5):369-375. doi: 10.1037/hea0000682. PMID: 31045419; PMCID: PMC6499487.

88. Choi Y, Mayer TG, Williams MJ, Gatchel RJ. What is the best screening test for depression in chronic spinal pain patients? Spine J. 2014 Jul 1;14(7):1175-82. doi: 10.1016/j.spinee.2013.10.037. Epub 2013 Nov 10. PMID: 24225008.
89. Chan CYY, Tsang HHL, Lau CS, Chung HY. Prevalence of depressive and anxiety disorders and validation of the Hospital Anxiety and Depression Scale as a screening tool in axial spondyloarthritis patients. Int J Rheum Dis. 2017 Mar;20(3):317-325. doi: 10.1111/1756-185X.12456. Epub 2014 Oct 7. PMID: 25293872.
90. Nipp RD, Fuchs G, El-Jawahri A, Mario J, Troschel FM, Greer JA, Gallagher ER, Jackson VA, Kambadakone A, Hong TS, Temel JS, Fintelmann FJ. Sarcopenia Is Associated with Quality of Life and Depression in Patients with Advanced Cancer. Oncologist. 2018 Jan;23(1):97-104. doi: 10.1634/theoncologist.2017-0255. Epub 2017 Sep 21. PMID: 28935775; PMCID: PMC5759817.
91. Mitchell AJ, Meader N, Symonds P. Diagnostic validity of the Hospital Anxiety and Depression Scale (HADS) in cancer and palliative settings: a meta-analysis. J Affect Disord. 2010 Nov;126(3):335-48. doi: 10.1016/j.jad.2010.01.067. Epub 2010 Mar 5. PMID: 20207007.
92. Sikora M, Verzak Ž, Matijević M, Včev A, Siber S, Musić L, Carek A. Anxiety and Depression Scores in Patients with Burning Mouth Syndrome. Psychiatr Danub. 2018 Dec;30(4):466-470. doi: 10.24869/psyd.2018.466. PMID: 30439807.
93. Malik R, Goel S, Misra D, Panjwani S, Misra A. Assessment of anxiety and depression in patients with burning mouth syndrome: A clinical trial. J Midlife Health. 2012 Jan;3(1):36-9. doi: 10.4103/0976-7800.98816. PMID: 22923978; PMCID: PMC3425147.
94. Burns JW, Day MA, Thorn BE. Is reduction in pain catastrophizing a therapeutic mechanism specific to cognitive-behavioral therapy for chronic pain? Transl Behav Med. 2012 Mar;2(1):22-9. doi: 10.1007/s13142-011-0086-3. PMID: 24073095; PMCID: PMC3717814.
95. Shi Z, Li S, Chen G. Assessing the Psychometric Properties of the Chinese Version of Ten-Item Personality Inventory (TIPI) Among Medical College Students. Psychol Res Behav Manag. 2022 May 16;15:1247-1258. doi: 10.2147/PRBM.S357913. PMID: 35603350; PMCID: PMC9121988.
96. Nunes A, Limpo T, Lima CF, Castro SL. Short Scales for the Assessment of Personality Traits: Development and Validation of the Portuguese Ten-Item Personality Inventory (TIPI). Front Psychol. 2018 Apr 5;9:461. doi: 10.3389/fpsyg.2018.00461. PMID: 29674989; PMCID: PMC5895732.
97. Thørrisen MM, Sadeghi T, Wiers-Jenssen J. Internal Consistency and Structural Validity of the Norwegian Translation of the Ten-Item Personality Inventory. Front Psychol. 2021 Aug 11;12:723852. doi: 10.3389/fpsyg.2021.723852. PMID: 34456829; PMCID: PMC8385139.
98. Tu TTH, Watanabe M, Suga T, Hong C, Takao C, Takenoshita M, Motomura H, Toyofuku A. Personality Traits in Burning Mouth Syndrome Patients With and Without a History of Depression. Front Psychiatry. 2021 Jul 29;12:659245. doi: 10.3389/fpsyt.2021.659245. PMID: 34393842; PMCID: PMC8358652.
99. Darnall BD, Sturgeon JA, Cook KF, Taub CJ, Roy A, Burns JW, Sullivan M, Mackey SC. Development and Validation of a Daily Pain Catastrophizing Scale. J Pain. 2017 Sep;18(9):1139-1149. doi: 10.1016/j.jpain.2017.05.003. Epub 2017 May 19. PMID: 28528981; PMCID: PMC5581222.
100. Cano A, Leonard MT, Franz A. The significant other version of the Pain Catastrophizing Scale (PCS-S): preliminary validation. Pain. 2005 Dec 15;119(1-3):26-37. doi: 10.1016/j.pain.2005.09.009. Epub 2005 Nov 17. PMID: 16298062; PMCID: PMC2679670.
101. Rogulj AA, Richter I, Brailo V, Krstevski I, Boras VV. CATASTROPHIZING IN PATIENTS WITH BURNING MOUTH SYNDROME. Acta Stomatol Croat. 2014 Jun;48(2):109-15. PMID: 27688354; PMCID: PMC4872800.
102. Walker NA, Sunderram J, Zhang P, Lu SE, Scharf MT. Clinical utility of the Epworth sleepiness scale. Sleep Breath. 2020 Dec;24(4):1759-1765. doi: 10.1007/s11325-020-02015-2. Epub 2020 Jan 14. PMID: 31938991.

103. Sap-Anan N, Pascoe M, Wang L, Grigg-Damberger MM, Andrews ND, Foldvary-Schaefer N. The Epworth Sleepiness Scale in epilepsy: Internal consistency and disease-related associations. Epilepsy Behav. 2021 Aug;121(Pt A):108099. doi: 10.1016/j.yebeh.2021.108099. Epub 2021 Jun 5. PMID: 34102473.
104. Frohnhofen H, Popp R, Willmann V, Heuer HC, Firat A. Feasibility of the Epworth Sleepiness Scale in a sample of geriatric in-hospital patients. J Physiol Pharmacol. 2009 Nov;60 Suppl 5:45-9. PMID: 20134038.
105. Damiani MF, Quaranta VN, Falcone VA, Gadaleta F, Maiellari M, Ranieri T, Fanfulla F, Carratù P, Resta O. The Epworth Sleepiness Scale: conventional self vs physician administration. Chest. 2013 Jun;143(6):1569-1575. doi: 10.1378/chest.12-2174. PMID: 23450315.
106. Adamo D, Sardella A, Varoni E, Lajolo C, Biasotto M, Ottaviani G, Vescovi P, Simonazzi T, Pentenero M, Ardore M, Spadari F, Bombeccari G, Montebugnoli L, Gissi DB, Campisi G, Panzarella V, Carbone M, Valpreda L, Giuliani M, Aria M, Lo Muzio L, Mignogna MD. The association between burning mouth syndrome and sleep disturbance: A case-control multicentre study. Oral Dis. 2018 May;24(4):638-649. doi: 10.1111/odi.12807. Epub 2018 Mar 13. PMID: 29156085.
107. Kirmizigil B, Demiralp C. Effectiveness of functional exercises on pain and sleep quality in patients with primary dysmenorrhea: a randomized clinical trial. Arch Gynecol Obstet. 2020 Jul;302(1):153-163. doi: 10.1007/s00404-020-05579-2. Epub 2020 May 15. PMID: 32415471.
108. Lee YH DDS, PhD, Chon S MD, PhD. Burning mouth syndrome in postmenopausal women with self-reported sleep problems. Cranio. 2020 Jul;38(4):221-232. doi: 10.1080/08869634.2018.1512549. Epub 2018 Aug 31. PMID: 30165803.
109. Lee GS, Kim HK, Kim ME. Relevance of sleep, pain cognition, and psychological distress with regard to pain in patients with burning mouth syndrome. Cranio. 2022 Jan;40(1):79-87. doi: 10.1080/08869634.2019.1681621. Epub 2019 Oct 24. PMID: 31648618.
110. Lopez-Jornet P, Lucero-Berdugo M, Castillo-Felipe C, Zamora Lavella C, Ferrandez-Pujante A, Pons-Fuster A. Assessment of self-reported sleep disturbance and psychological status in patients with burning mouth syndrome. J Eur Acad Dermatol Venereol. 2015 Jul;29(7):1285-90. doi: 10.1111/jdv.12795. Epub 2014 Oct 28. PMID: 25351854.
